# Synthesis and Crystal Structure of Adamantylated 4,5,6,7-Tetrahalogeno-1*H*-benzimidazoles Novel Multi-Target Ligands (Potential CK2, M2 and SARS-CoV-2 Inhibitors); X-ray/DFT/QTAIM/Hirshfeld Surfaces/Molecular Docking Study

**DOI:** 10.3390/molecules28010147

**Published:** 2022-12-24

**Authors:** Jolanta Natalia Latosińska, Magdalena Latosińska, Andrzej Orzeszko, Jan Krzysztof Maurin

**Affiliations:** 1 Faculty of Physics, Adam Mickiewicz University, Uniwersytetu Poznańskiego 2, 61-614 Poznań, Poland; 2 Institute of Chemistry, Warsaw University of Life Sciences, 159C Nowoursynowska St., 02-787 Warsaw, Poland; 3 National Medicines Institute, Chełmska 30/34, 00-750 Warsaw, Poland; 4 National Centre for Nuclear Research, Andrzeja Sołtana 7, 05-400 Otwock-Świerk, Poland

**Keywords:** multi-target ligands, CK2 inhibitor, M2 blocker, SARS-CoV-2 inhibitor, synthesis, structure, interactions pattern, molecular docking

## Abstract

A series of new congeners, 1-[2-(1-adamantyl)ethyl]-1*H*-benzimidazole (AB) and 1-[2-(1-adamantyl)ethyl]-4,5,6,7-tetrahalogeno-1*H*-benzimidazole (Hal=Cl, Br, I; tClAB, tBrAB, tIAB), have been synthesized and studied. These novel multi-target ligands combine a benzimidazole ring known to show antitumor activity and an adamantyl moiety showing anti-influenza activity. Their crystal structures were determined by X-ray, while intermolecular interactions were studied using topological Bader’s Quantum Theory of Atoms in Molecules, Hirshfeld Surfaces, CLP and PIXEL approaches. The newly synthesized compounds crystallize within two different space groups, P-1 (AB and tIAB) and P2_1_/c (tClAB and tBrAB). A number of intramolecular hydrogen bonds, C−H⋯Hal (Hal=Cl, Br, I), were found in all halogen-containing congeners studied, but the intermolecular C−H⋯N hydrogen bond was detected only in AB and tIAB, while C−Hal⋯π only in tClAB and tBrAB. The interplay between C−H⋯N and C−H⋯Hal hydrogen bonds and a shift from the strong (C−H⋯Cl) to the very weak (C−H⋯I) attractive interactions upon Hal exchange, supplemented with Hal⋯Hal overlapping, determines the differences in the symmetry of crystalline packing and is crucial from the biological point of view. The hypothesis about the potential dual inhibitor role of the newly synthesized congeners was verified using molecular docking and the congeners were found to be pharmaceutically attractive as Human Casein Kinase 2, CK2, inhibitors, Membrane Matrix 2 Protein, M2, blockers and Severe Acute Respiratory Syndrome Coronavirus 2, SARS-CoV-2, inhibitors. The addition of adamantyl moiety seems to broaden and modify the therapeutic indices of the 4,5,6,7-tetrahalogeno-1*H*-benzimidazoles.

## 1. Introduction

The recent endeavors in the field of drug design are aimed at discovering multi-target ligands, which could be an alternative to the known mono-targeted drugs, effective for the treatment of multi-factorial inflammation or inhibition of carcinogenic processes [1]. Some substructures like benzimidazole or adamantine, capable of binding with high affinity to multiple receptors and enzymes and modulating pathways associated with the inflammatory or carcinogenic processes, are privileged. Thus, they play a key role in drug design and nanotechnology [2,3].

Recently, a variety of novel benzimidazole derivatives have been synthesized and screened for their various anticancer [2,4,5,6,7], antitumor [8], antiviral [8,9,10], hormone antagonist [11], anti-HIV [12], anthelmintic and anti-protozoal [13], anti-bacterial [14,15], anti-mycobacterial [16], anti-hypertensive [17], anti-inflammatory [18], analgesic [19], anxiolytic [20], coagulant/anticoagulant [21], anti-allergic [22], anti-HBV [23], anti-oxidant [24] and anti-diabetic [25] activities. 

Adamantane derivatives blocking the proton-selective ion channel of Matrix 2, M2, viral protein (essential for viral reproduction) are well known to act as prophylactic agents against many different types of influenza, including avian influenza (Asian influenza) and influenza type A [26,27,28,29,30] excluding H1N1 or H5N5, which are adamantyl resistant [31,32]. The importance of the adamantyl group, playing multiple roles during the viral replication cycle, has been recognized during the design of agents effective for the treatment of hepatitis B and C [33], neurodegenerative disorders (Alzheimer’s, Huntington’s and Parkinson’s diseases) [34,35], multiple-sclerosis [36], depression [36], age-related cataract [37], cancer [38,39,40,41], diseases characterized by pathological immune suppression [42], hemochromatosis (in development), malaria (in clinical trials) and type 2 diabetes (in the clinical phase) [43], but also in anti-herpes [44], anti-HIV [45,46,47,48], anti-HPV1 [49], analgetic and anti-convulsant drugs [28]. Moreover, amantadine has been recently shown to inhibit Severe Acute Respiratory Syndrome Coronavirus 2 (SARS-CoV-2) replication in Vero E6 cells [50], while its derivative memantine (used to slow Alzheimer disease progression) has been the starting point to obtain the aminoadamantane nitrate compound, NMT5, which has potential to turn SARS-CoV-2 against itself [51]. 

The aim of our study was to synthesize a set of congeners taking advantage of both moieties (4,5,6,7-tetrahalogeno-1*H*-benzimidazole ring and adamantyl skeleton). A combination of two substructures, one showing antitumor activity and the other showing anti-influenza activity, has been prompted by the observation that some oncolytic viruses destroy tumor cells [52,53], and that impaired T-Cell response, elevated level of cytokines or long inflammation (caused by influenza or COVID-19 infection) may facilitate tumorigenesis and cancer progression. Such a combination of moieties in one compound ensures attraction to both polar and non-polar environments, i.e., amphiphilic properties. The introduction of the nonpolar adamantyl to 4,5,6,7-tetrahalogeno-1*H*-benzimidazoles enhances their lipophilicity, which is a key property in the transport processes inside the body. Incorporation of the -CH_2_CH_2_- chain which separates adamantyl from benzimidazole moiety should add conformational flexibility to the structure, highly required for the biological activity (e.g., anti-HIV [54]). 

**Figure 1:**
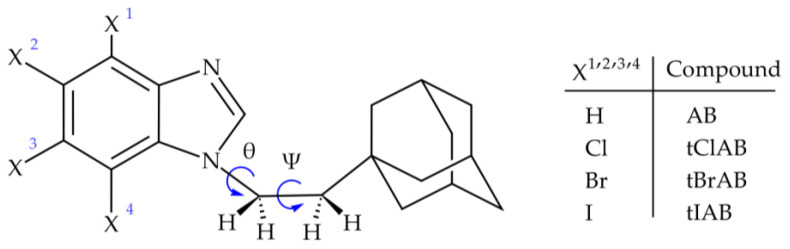
Structural formula of adamantylated 4,5,6,7-tetrahalogeno-1*H*-benzimidazoles, (torsion angles: ψ = ∠C(2)N(1)C(10)C(11) and θ = ∠N(1)C(10)C(11)C(12)).

We synthesized four such compounds: 1-[2-(1-adamantyl)ethyl]-1*H*-benzimidazole (AB) or 2-(1-adamantyl)-4,5,6,7-tetrahalogeno-1*H*-benzimidazoles (X=Cl, Br, I; tClAB, tBrAB tIAB, respectively), Figure 1. 

Because the conformation and interplay of the intra- and intermolecular interactions are crucial from the biological point of view (protein–ligand binding), we focused our study on the structural aspects of the newly synthesized congeners. While the molecular conformation is often determined by electrostatic interactions, the molecular recognition is governed mostly by weak non-covalent interactions (hydrogen bonds, van der Waals forces, steric interactions, π-effects and hydrophobic effects). The X-ray combined with modern approaches, including the Density Functional Theory (DFT) calculations augmented with an empirical dispersion, Bader’s Quantum Theory of Atoms in Molecules (QTAIM) [55,56], Spackman’s Hirshfeld surfaces [57,58], Politzier’s Electrostatic Potential (ESP) [59], CLP (atom–atom) [60] and Pixel (quantum chemistry-based) [61], were used for investigation of the binding propensity in solid. They provide a deep insight into three-dimensional crystalline packing at the atomic and molecular levels and reveal the nature of the intermolecular interactions [62,63,64,65].

The potential dual inhibitor role of this new class of compounds was checked and the mutual influence of ethyl-adamantyl and 4,5,6,7-tetrahalogeno-1*H*-benzimidazoles was analyzed from the point of view of topology of the interaction pattern and the molecular recognition. The structural features in solid were used to predict the propensity of congeners studied to bind with three kinds of targets: human casein kinase 2 (CK2), virus proton channel (M2) and SARS-CoV-2 spike protein. The specific molecular conformation determined by X-ray was treated as the initial point for further studies performed in silico. Molecular docking was used to characterize the specificity of the binding site in M2, CK2 and SARS-CoV-2 targets, for the prediction of possibilities and strength of the binding of new ligands with targets and forecasting the therapeutic effectiveness of synthesized compounds in silico. We anticipate that our study will be helpful in outlining the directions of synthesis of new multi-target ligands that would be promising leads for medicinal chemistry optimization with the ultimate goal of developing a broad spectrum of effective drugs.

## 2. Results and Discussion

### 2.1. Crystal Packing Analysis

The X-ray data for 1-[2-(1-adamantyl)ethyl]-1*H*-benzimidazole (AB) and three 1-[2-(1-adamantyl)ethyl]-4,5,6,7-tetrahalogeno-1*H*-benzimidazoles (X=Cl, Br or I; tClAB, tBrAB and tIAB), Figure 1, collected at room temperature, RT, are listed in Table 1 and Tables S1–S20 (Supplementary).

**Figure 2:**
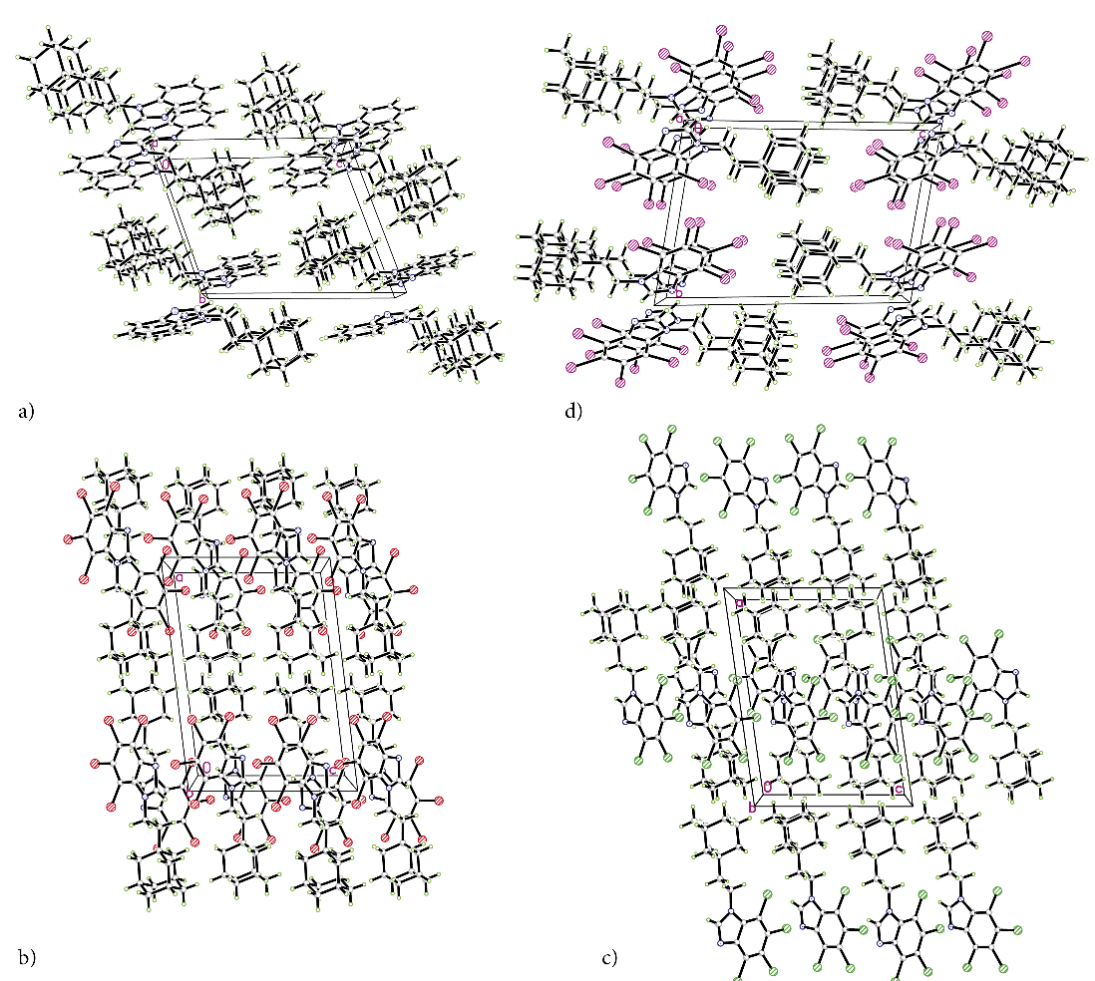
The packing of the crystal structures of adamantylated 4,5,6,7-tetrahalogeno-1*H*-benzimidazoles. (**a**) AB, (**b**) tClAB, (**c**) tBrAB and (**d**) tIAB.

The refinement with anisotropic displacement parameters for all non-hydrogen atoms was performed, with the final *R*-factors (residual factors) listed in Table 1. Measurements were performed with Cu K_α_ radiation, which leads to high absorption that must be taken into account to the highest possible accuracy. High peaks and holes in the differential map in the vicinity of bromine and iodine atoms were expected. A slightly higher *R*-factor for tBrAB results from high bromine content, which makes this compound in solid state opaque to X-rays to a high degree. The crystalline packings are shown in Figure 2a–d. 

The atomic positions, bond lengths and angles for AB, tClAB, tBrAB and tIAB are collected in Tables S1–S20, respectively. Newly synthesized congeners AB, tClAB, tBrAB and tIAB are isostructural in pairs (AB and tIAB versus tBrAB and tClAB), Table 1. The interlayer distances (defined as the distances between layers containing neighbouring stacking molecules) are:

3.214 Å (H; AB) < 3.315 Å (I; tIAB) and 3.451 Å (Cl; tClAB) < 3.534 Å (Br; tBrAB)(1)

and reflect the division into P-1 and P2_1_/c space groups upon crystallization.

Examination of the deviations from the least-square planes through the benzimidazole rings indicates that heterocyclic 4,5,6,7-tetrahalogeno-1*H*-benzimidazole skeletons are almost perfectly planar in all compounds studied (within 3σ^2^). Even halogen atoms, despite spatial crowding, lie in the plane of benzimidazole (with the exception of iodine at X_3_ position, which is deviated by 3° relative to the benzimidazole plane). It suggests a reasonable degree of conjugation over the entire benzimidazole unit.

Overall conformation of the molecules in the set of congeners is nearly the same. It can be described by the dihedral angle between the benzimidazole ring and ethyl-adamantyl moiety, ∠C(2)N(1)C(10)C(11), which decrease in the order:

105.16° (H; AB) > 97.30° (Cl; tClAB) > 96.24° (Br; tBrAB) > 85.09° (I; tIAB)(2)

reflecting the increase in the van der Waals atomic radius of the halogen atom, and by the dihedral angle between adamantyl and ethyl-adamantyl moiety, ∠N(1)C(10)C(11)C(12), which decrease in the order:

177.64° (Cl; tClAB) > 176.86° (Br; tBrAB) > 176.23° (I; tIAB) > 172° (H; AB)(3)

reflecting a decrease in halogen’s electronegativity (decreasing negative inductive effect) and conjugation (decreasing positive mesomeric effect). The angle changes are subtle because the adamantyl moiety is bulky and easily forms weak contacts; thus, it strongly hinders rotation.

Detailed analysis of the nature of contributions to total lattice energy derived using the CLP (atom–atom; evaluation based on cell dimensions and atomic nuclear coordinates) and Pixel (quantum chemistry-based; calculations based on the ab initio molecular orbital calculations as numerical integrals over a large number of electron-density units) techniques has revealed that the major contribution comes from dispersion, while the electrostatic terms (coulombic and polarization) are smaller and cannot be cancelled by the repulsion, Table 2 and Table 3.

**Figure 3:**
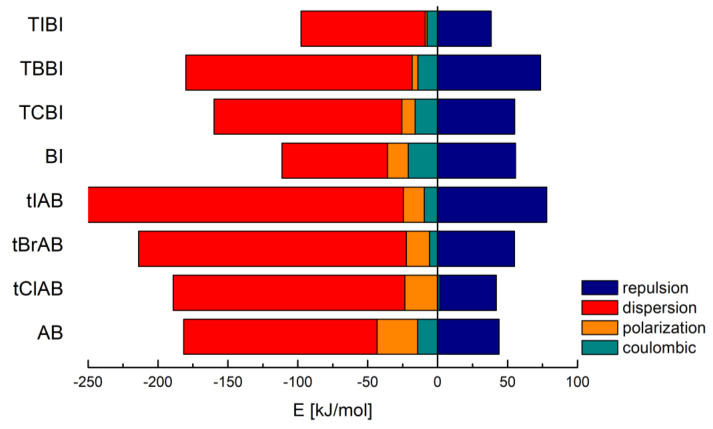
Contributions to the total lattice energy broken down into a few different basic interaction types in adamantylated 4,5,6,7-tetrahalogeno-1*H*-benzimidazoles, 1*H*-benzimidazole (BI) and 4,5,6,7-tetrahalogeno-1*H*-benzimidazoles (TIBI, TBBI, TCBI).

CLP analysis indicates that the dispersion and repulsion terms increase but the polarization one decreases with increasing van der Waals radius of halogen atoms. CLP marginalizes the role of electrostatic interactions, especially coulombic ones, Table 2 and Figure 3.

Much more reliable Pixel analysis indicates that in the set of congeners, the repulsion, polarization and coulombic components steadily increase with increasing van der Waals radius/decreasing electronegativity of halogen, Table 3, which resembles the structure stretching effect observed for 4,5,6,7-tetrahalogeno-1*H*-benzimidazoles. Spackman’s approach [57,58] leads to the smaller values, but the trend remains the same, Table 4.

The intermolecular interactions in lattice are dominated by dispersion forces, which for larger and heavier molecules of adamantylated 4,5,6,7-tetrahalogeno-1*H*-benzimidazoles are much stronger than in the previously studied 4,5,6,7-tetrahalogeno-1*H*-benzimidazoles.

### 2.2. Interactions Pattern in the Solid State

#### 2.2.1. Distribution and Types of Interactions—3D HS/2D FP

**Figure 4:**
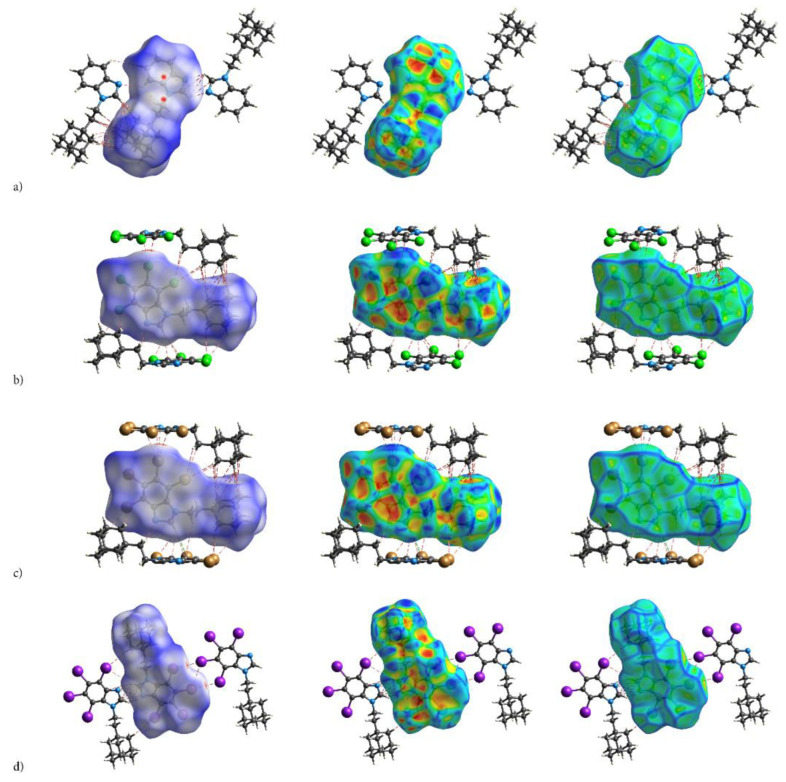
The crystal structures of adamantylated 4,5,6,7-tetrahalogeno-1*H*-benzimidazoles with d_norm_ (left; colour scale: negative (red) to positive (blue) values), shape index (centre; concave (red) and convex (blue) regions) and curvedness (right; the planar (green) and curved (blue edge) regions) over total 3D Hirshfeld surface: (**a**) AB, (**b**) tClAB, (**c**) tBrAB and (**d**) tIAB.

Some findings important for evaluation of biological activity come from the analysis of the 3D Hirshfeld Surfaces (3D HS). The combined analysis of 3D HS and 2D molecular fingerprints (2D FP) derived from 3D HS; which summarize the distribution of interactions of the molecule with its environment and electrostatic potential (ESP), which play a role in identification of electrostatic interactions between molecules; delivers an insight into the mix of intermolecular interactions (mainly H⋯H, N⋯H/H⋯N, C⋯H/H⋯C, X⋯H/H⋯X, X⋯X; X=Cl, Br, I) in the crystals. The 3D HS with the normalized contact distance d_norm_, shape-index and curvedness, mapped over this surface for all congeners are shown in Figure 4a–d, while quantitative parameters are collected in Table 5.

**Figure 5:**
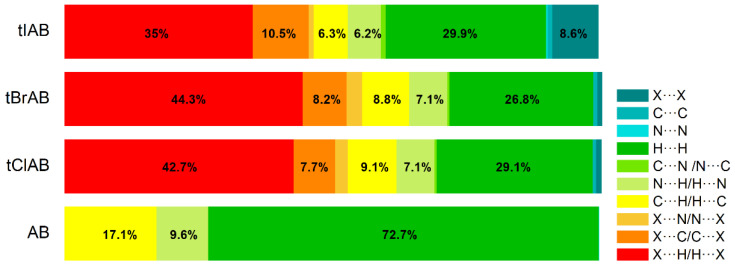
Percentage contributions to the 3D Hirshfeld surface area from the various intermolecular contacts in the set of compounds studied.

Hirshfeld surface analysis indicates that H⋯H, X⋯H/H⋯X (X=Cl, Br or I), N⋯H/ H⋯N and C⋯H/ H⋯C contacts are the most significant contributors from among the interacting atoms, Table 5 and Figure 5. The most significant contribution to these interactions is brought by the hydrogen and halogen atoms. The decrease in the contribution from halogens in favor of contribution from the hydrogens occurs with increasing electronegativity. The enrichment ratio, E_XY,_ of the main intermolecular contacts, Table 6, reveals privileged and disfavored contacts between two atomic species, X and Y. The E_XY_ is larger than one for a pair of atoms with greater propensity to form contacts. The H⋯H contacts appear with enrichment ratio close to unity only in tIAB. The propensity of C⋯X/X⋯C contacts is enriched in all halogenated compounds. The N⋯H/H⋯N and N⋯N contacts are significantly enriched only in tIAB and AB, whereas X⋯H/H⋯X appear with significantly enrichment ratio only in tClAB and tBrAB.

**Figure 6:**
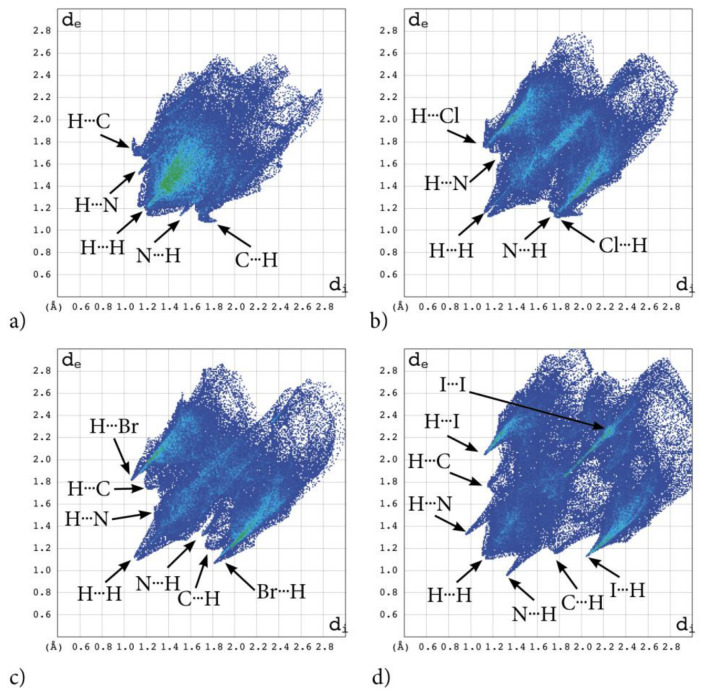
2D molecular fingerprints of the interaction pattern in adamantylated 4,5,6,7-tetrahalogeno-1*H*-benzimidazoles: (**a**) AB, (**b**) tClAB, (**c**) tBrAB and (**d**) tBrAB. Points on the plot corresponding to small contributions to the 3D HS surface are coloured dark blue, those with moderate contribution are coloured green and those with the greatest contribution are coloured red. All four fingerprint plots are coloured in the same scale.

The most significant contribution is brought by very weak H⋅⋅⋅H interactions, which cover as much as 72.7, 29.1, 26.8, 29.9% of the total 3D HS for AB, tClAB, tBrAB and tIAB, respectively. In the 2D FP, they are reflected by the cloud of scattered points for AB and one common much broadened spike along the d_i_ = d_e_ line for tClAB, tBrAB and tIAB, Figure 6. The neighbouring ethyl-adamantyl moieties bind together in the structure to form a kind of “chain”. This stabilizing contribution mainly comes from weak CH⋯HC London dispersion and recoupling of covalent CH bonds with H⋯H bonds. The enrichment ratio, 2D scattering of FPs and the higher melting points of halogenated compounds suggest that, while these interactions are the most common, they are certainly neither privileged nor the strongest in the structure.

A substantial difference between the intermolecular patterns in the pairs AB, tIAB, and tBrAB, tClAB is the occurrence of the strong hydrogen bonds donated by CH and accepted by N atoms in the former and their lack in the latter pair. (This effect is reflected in the melting temperatures and decomposition upon melting, Table 1). The hydrogen bonds, HB, are indicated by intense red areas in 3D HS, Figure 4.

For AB and tIAB, intense red areas in the 3D HS near N and H at C(2) represent the lowest values of d_norm_ (−0.039 and −0.252 a.u. for AB and tIAB, respectively) and the contact patches (the flattened regions of the 3D HS with curvedness and shape-index mapped over this surface, Figure 4). These interactions cover a small area of 9.6 and 6.2% of the total 3D HS for AB and tIAB, respectively. In 2D FP d_e_/d_i_ plots derived from 3HS, Figure 6, the N⋯H/H⋯N contacts are represented by symmetric spikes (‘‘wings’’), sharp and small at d_e_ + d_i_~2.4 Å and 2.65 Å for tIAB and AB, respectively. They confirm the presence of C(2)-H⋯N(1) hydrogen bonds, which are long (R_C⋯N_ = 3.408 Å) and nonlinear (<CHN = 129.57°), in AB. These bonds form a dimer with benzimidazole rings laying in parallel planes distanced by 1.584 Å (a tangled staircase ribbon) and adamantyl moieties laying on both sides of this dimer. In tIAB, isostructural with AB, two hydrogen bonds C(2)-H⋯N(1) are also nonlinear (<CHN = 128.55°) but much shorter (R_C⋯N_ = 3.095 Å), thanks to a much smaller (0.66 Å) spatial separation of molecules in the dimer. These bonds are stronger but competitive to two van der Waals contacts of 3.724 Å linking I(3)⋯I(4) atoms in tIAB (d_norm_ = −0.116 a.u.), which form another kind of dimer. These double dimers in tIAB form a nearly flat ribbon, with adamantyl moieties residue on both sides of this ribbon, Figure 2. The percentage contribution of I⋯I to 3D HS is quite high (8.6%). In a 2D FP plot, they are visualized by a sharp and narrow single spike near the midsection d_e_ = d_i_ line at d_e_ + d_i_~3.7 Å. 

For tClAB and tBrAB, the C(2)-H⋯N interactions are replaced by the C(2)-H⋯X(3) ones, with halogen (Cl or Br) playing the role of acceptor, instead of nitrogen. These bonds, C(2)-H⋯Cl(3) of 3.984 Å and C(2)-H⋯Br(3) of 3.945 Å, respectively, are much longer and weaker than C(2)-H⋯N. The X⋯H/H⋯X (X=Cl or Br) contacts are represented in 2D FP by wide sharp spikes at d_e_ + d_i_~2.95 Å for tClAB and tBrAB, i.e., shorter than d_e_ + d_i_~3.15 Å for tIAB. They cover as much as 42.7 and 44.3% of the total 3D HS for tClAB and tBrAB, respectively; more than 35% for tIAB. In tClAB and tBrAB, the main motif forcing the perpendicular orientation of the molecule’s interaction is indicated by intense red areas of the lowest value of d_norm_ (−0.127 and −0.180 a.u., respectively) localized near Cl(3) and Br(3) regions matching the relevant areas of the highest value of d_norm_ (1.495 and 1.487 a.u., respectively). It describes C-Cl⋯π and C-Br⋯π stacking interactions (the most common noncovalent interaction motifs in synthetic compounds). The X⋯C/C⋯X contacts are represented by the widest sharp spikes, covering as little as 7.7, 8.2, and 10.5% of the total 3D HS for tClAB, tBrAB and tIAB, respectively. The X⋯X interactions bring a small 8.6% contribution for tIAB, but only about 1% for tClAB and tBrAB, and are reflected by the cloud of concentrated points along the upper region of d_i_ = d_e_ line for tClAB, tBrAB and tIAB. 

The C⋯H/H⋯C interactions are represented by wider spikes at d_e_ + d_i_~2.8 Å for AB, d_e_ + d_i_~2.9 Å for tClAB and d_e_ + d_i_~3.0 Å for tBrAB and tIAB and cover 17.1, 9.1, 8.8 and 6.3% of the total 3D HS for AB, tClAB, tBrAB and tIAB, respectively. On the 3D HS, they are poorly visible but on the shape index mapped over 3D HS, they can be easily recognized as intense red areas indicated by the C-H bonds. These contacts describe the C⋯H/H⋯C bonds that are involved in stabilising the ethyl-adamantyl conformation and binding adjacent ethyl-adamantyls to each other. In AB, these interactions link 1*H*-benzimidazole ring to ethyl-adamantyl and 1*H*-benzimidazole rings to each other. In tClAB and tBrAB, the main motif forcing the perpendicular orientation of the molecule interaction is indicated by intense red areas of the lowest value of d_norm_ (−0.127 and −0.180 a.u., respectively) localized near Cl(3), and Br(3) regions matching the relevant areas of the highest value of d_norm_ (1.495 and 1.487 a.u., respectively) are C-Cl⋯π and C-Br⋯π stacking interactions (the most common noncovalent interaction motifs in synthetic compounds). They can be identified via blue-red triangles in the shape index surfaces, Figure 4. In 2D FP, the X⋯C/C⋯X (X=Cl, Br, I) contacts are represented by the widest sharp spikes covering as little as 7.7, 8.2, and 10.5% of the total 3D HS for tClAB, tBrAB and tIAB, respectively.

The contribution of X⋯X (X=Br, Cl) contacts is marginal (about 1%). In 2D FP, they are reflected by a small cloud of concentrated points along the upper region of the d_i_ = d_e_ line. The fact that the most prominent short range intermolecular contacts responsible for the packing arrangement and formation of the 3D network structure in the crystals of congeners are weak contacts partially explains the dominance of the intermolecular interactions in the crystals by dispersion.

#### 2.2.2. Electrostatic Nature of Interactions—ESP

**Figure 7:**
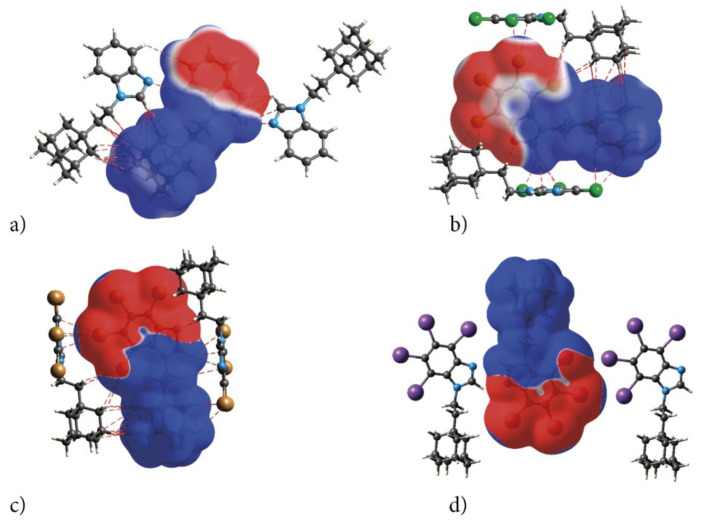
Electrostatic complementarity upon crystal packing in adamantylated 4,5,6,7-tetrahalogeno-1*H*-benzimidazoles: electrostatic potential mapped onto the Hirshfeld surfaces of symmetry-related neighbouring molecules (**a**) AB, (**b**) tClAB, (**c**) tBrAB and (**d**) tIAB. Color scale is −0.01 (red) to 0 (white) to 0.01 a.u. (blue). The negative ESP (repulsive) on both sides of the molecule occupy a C-shaped region, symmetrical on the top and bottom sides of the molecule.

The electrostatic nature of the interactions, that is, the match of positive and negative regions of the molecular surfaces (electrostatic complementarity), is well pronounced in ESP. The positive (blue) regions of ESP are clearly separated from the negative (red) regions by white zero-potential lines, Figure 7. The highly positive region (relative absence of electrons) of ESP is localized on H atoms of ethyl-adamantyl moiety and CH from 1*H*-benzimidazole ring, while the negative one (abundance of electrons) is on almost the whole 1*H*-benzimidazole ring. This separation well describes charge transfer direction in the molecule, from neutral adamantyl to electron-withdrawing halogens. But these properties of the ESP also indicate the preferred interaction sides of the molecules. As actually realized in the crystal, three halogen atoms and unsubstituted nitrogen act as acceptors of intermolecular hydrogen bonds, while ethyl-adamantyl carbons and CH from 1*H*-benzimidazole ring act as donors.

The ESP point to the electrostatic nature of these bonds. Additionally, the pattern of positive and negative electrostatic potential regions on the molecular surface of the congeners helps to understand formation of the dimers in the crystals of congeners—the negatively charged 4,5,6,7-tetrahalogeno-1*H*-benzimidazole rings are alternated with the positive ethyl-adamantyl groups (they perfectly match each other).

The variation of the positive and negative values of the ESP is reflected by the values of average V_S_^−^ and V_S_^+^ potentials, the highest V_S_^min^ and V_S_^max^ potentials and the smallest variances, σ_+_^2^ and σ_−_^2^, Table 7. 

The absolute values of V_S_^min^ are much higher for tIAB, tClAB and tBrAB than for TIBI, TCBI and TBBI, respectively, which means that the long-range attraction of electrophiles will be significantly enhanced. The values of V_S_^max^ are slightly lower for tIAB, tClAB and tBrAB than for TIBI, TCBI and TBBI, respectively; thus, the long-range attraction of nucleophiles will be subtly weakened. The same effect of the enhancement of the long-range attraction of electrophiles and weakening of the long-range attraction of nucleophiles occurs for tBrAB. 

The change in the degree of balance between the positive and negative potentials on the ESP, η_S_, for tIAB, tClAB and tBrAB is evident: η_S_ is much higher than for TIBI, TCBI and TBBI. Moreover, η_S_ is very small for AB, but close to the maximum possible value (0.25) for tIAB, tClAB and tBrAB. So high values suggest that halogenated congeners, due to the addition of the neutral ethyl-adamantyl, are more balanced and able to interact to a similar extent through positive or negative potentials (a number of hydrogen bond donors and acceptors should be equal). However, a comparison of the parameters σ_+_^2^ and σ_−_^2^, which are the measure of the variability within the positive and negative regions of the surface potential, suggests that only tIAB should be as good a hydrogen bond acceptor as a donor. The other congeners are predicted to be slightly better donors than acceptors. This effect is reflected in the crystalline structure—the strongest hydrogen bonds are formed in tIAB. The η_S_σ_T_^2^ parameter, which shows how strongly a molecule interacts with the other ones of the same kind, is well correlated with melting points, the total lattice energy and strength of the intermolecular interactions for the compounds studied. Moreover, it takes lower values for tIAB, tClAB and tBrAB than for TIBI, TCBI and TBBI, which also indicates a significant difference in electrostatic interactive tendencies. The magnitude of π, which describes the average deviation from the mean surface potential and can be treated as a measure of local polarity of a molecule, increases in the order AB < tClAB ≈ tIAB < tBrAB (i.e., the same as dipole moments). The lipophilicity of the ligand, a key factor for the transport inside the body, and its solubility, which are important issues in the field of drug discovery, are both related to the ligand’s polarity, which is predicted to be the highest for tBrAB. 

The above characteristics are important from the docking point of view. In the protein pocket, the ligand should adopt an orientation that will bring its charged fragments into matching attractive interaction with positive and negative potential pattern. Thus, the structural requirement for the interaction with the protein can be inferred from the potential map of the actual active ligand. It can be used as a criterion for the ability of newly constructed ligands to mimic the same reactive pattern or for the choice of the correct orientation of the ligand inside the pocket, which is of particular importance in the case of its elongated shape (e.g., ionic channel).

#### 2.2.3. Characterization of the Strength of the Interactions—QTAIM

**Figure 8:**
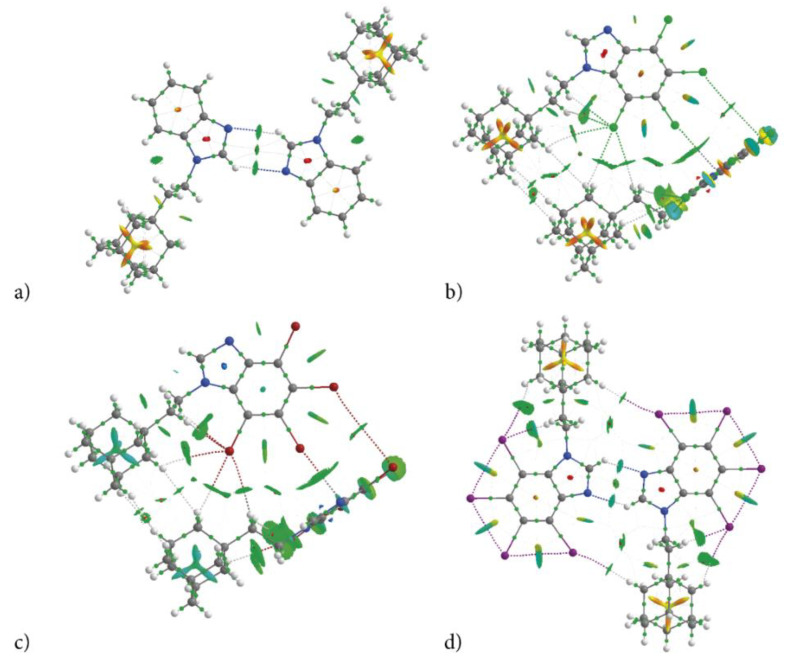
Molecular graph of dimers cut of crystalline structure of congeners (bond critical point, BCP—small green points, ring critical point, RCP—small red points) (**a**) AB, (**b**) tClAB, (**c**) tBrAB and (**d**) tIAB. The isosurface of reduced density gradient surface (RGD = 0.5) with sign(λ_2_)ρ_BCP_ is mapped over the surface visualize interacting regions. Color scale is −0.03 (blue) to 0.03 a.u. (red).

The Quantum Theory of Atoms in Molecules (QTAIM) methodology was used for characterization of strength of the inter- and intra-molecular interactions revealed by 3D HS and 2D FP that hold a specific molecular conformation and determine crystal packing. The classical approach, i.e., the calculation of the energy difference between a dimer and two monomers, is useless due to the multiple bonds of different type linking two molecules in a dimer. The topological descriptors of electron density (electron density at bond critical point ρ(r), its Laplacian Δρ(r), the total electron energy density H_BCP_(r), and its components the kinetic electron energy density G_BCP_(r), and the potential electron energy density V_BCP_(r)) and the strength of all intra- and intermolecular bonds and contacts calculated according to Espinosa−Molins−Lecomte (E_HBE_), Matta (E_HBM_)_,_ Emamian (E_HBEM_), Afonin (E_HA_) and Kuznetsov (E_XXK_) approaches, are collected in Table 8. The molecular graphs of dimers cut of crystalline structure with bond critical points (BCPs), ring critical points (RCPs) and reduced density gradient isosurfaces with sign(λ_2_)ρ_BCP_ mapped over the surface, are shown in Figure 8. The dimer’s formation does not affect the intramolecular bonds formed in monomers, but it introduces additional ones.

The total energy density H_BCP_ at each BCP is small but positive, which is indicative of the closed-shell interaction. Both Δρ_BCP_ > 0 and H_BCP_ > 0 allow classification of these interactions as weak. All of them are non-covalent (−G_BCP_/V_BCP_ > 1) and thermodynamically feasible (the bonding energy E_HB_ < 0). The strength of intermolecular interactions is partially reflected by ρ_BCP_ (0.003–0.017 a.u.), Δρ_BCP_ (0.012–0.044 a.u.), but also by the negative sign(λ_2_)ρ_BCP_ at all bond critical points (BCPs). For the estimation of the strength of recognized hydrogen bond interactions, Espinosa−Molins−Lecomte, Matta, Emimem and Afonin approximations (the equations are listed in the paragraph 3.8) were applied, Table 8. The differences between the energies obtained using the Espinosa−Molins−Lecomte and Matta methods are rather subtle and do not exceed 0.5 kJ/mol per interaction. (Only in a few cases of strong dispersive interactions, they reach as much as 2.0 kJ/mol.) Emimem’s formula leads to smaller values, but the trend remains the same. However, for very weak hydrogen bonds, (C-H⋯X, X=Cl, Br, I) in our compounds, Emimem’s equation leads to the repulsion instead attraction, which is the result of the extrapolation of an empirical formula obtained for a different class of compounds having stronger interactions. Since Emimem’s analysis did not take into account such weak bonds as those found in the tIAB structure, the application of the empirical formula proposed by him leads to the repulsion instead of attraction, although the other indices (ρ_BCP_, Δρ_BCP_, H_BCP_/ρ_BCP_, |V_BCP_|/Δρ_BCP_, Δρ_NCP_) confirm the presence of the hydrogen bonds. Afonin’s scaled formula E_HBA_(|V|) allows obtaining energies, which seems reasonable, but his formula E_HB_(ρ_BCP_) results in repulsion instead of attraction. This clearly indicates that the linearly scaled equations are not universal, and their validity should be restricted to the types of the interactions and molecular systems to which they were deduced. Otherwise, it may lead to incorrect results. However, it should be emphasized that all these approaches (Espinosa−Molins−Lecomte, Matta, Emimem, Afonin and others) differ in scaling factors and the results are linearly dependent. Thus, a comparison of the relative strength of interaction for a set of compounds calculated using the same formula is independent of the used formulae. Even a coarse Espinosa−Molins−Lecomte formula, which overestimates the bond strength, properly evaluates the relationship between strengths of the interactions for a set of compounds.

For the estimation of the strength of halogen-halogen contacts, Kuznetsov formulae were applied, but Espinosa−Molins−Lecomte or Matta formulae also properly evaluate the relationship between strengths of the X⋯X interactions.

According to the total strength of the intermolecular interactions, the congeners can be ordered as follows: tIAB (−99.2 kJ/mol) >> tClAB (−45.4 kJ/mol) ≅ tBrAB (−46.7 kJ/mol) >> AB (−28.8 kJ/mol), which is correlated with the ordering by global packing (**1**), dispersion and total lattice energy, Table 3. QTAIM analysis confirms that, despite a significant difference in crystalline packing of AB, tIAB and tCAB, tBrAB, the same type of interactions keeps identical conformations of molecules of all four congeners, Figure 8a–d. Solely on the basis of the H⋅⋅⋅X distances and ∠CHX angles, it is evident that in halogen-containing compounds (tIAB, tBrAB and tClAB), the intramolecular hydrogen bonds donated by the ethyl-adamantyl group and accepted by the halogen atoms, C-H⋯X, are formed. The molecular conformation is stabilized by the sets of C-H⋯X of different strength (two of −1.71 and −11.16 kJ/mol in tIAB.; three of −2.36, −5.25 and −8.01 kJ/mol in tBrAB.; −2.10, −4.99 and −8.27 in tClAB). In AB and tIAB, the conformation is stabilized by C-H⋯H-C dispersive interactions of −9.79 and −9.80 kJ/mol, respectively. 

The strongest interactions in AB are two C-H⋯N intermolecular hydrogen bonds of −4.59 kJ/mol forming a dimer*,* Figure 8a. The sign(λ_2_)ρ_BCP_ of this interaction is equal to −0.00004 a.u.; thus, it can be classified as very weak. In tIAB, a dimer is formed by two C-H⋯N hydrogen bonds of −10.96 kJ/mol, Figure 8d. They are slightly stronger, as the sign(λ_2_)ρ_BCP_ is equal to −0.00016 a.u. Two weak intermolecular C-H⋯X hydrogen bonds of −2.10 kJ/mol assist in the dimer formation in tIAB. Intramolecular X⋯X van der Waals contacts in tIAB are very strong, of about −9.36, −9.37 and −9.38 kJ/mol. 

Different kind of dimers are formed in tClAB and tBrAB with the use of a set of interactions (CH⋯X, H⋯H and X⋯π) linking two 4,5,6,7-tetrahalogeno-1*H*-benzimidazole rings and three H⋯H contacts linking adamantyl moieties, Figure 8b,c. The numerous H⋯H contacts are of −2.10 to −3.15 kJ/mol in tClAB and of −1.44 to −3.41 kJ/mol in tBrAB. The CH⋯X bonds are of −2.10 and −2.23 kJ/mol in tClAB and −1.97 and −2.36 kJ/mol in tBrAB. The X⋯π of −6 kJ/mol in tClAB and −6.73 kJ/mol in tBrAB are the strongest; X⋯X contacts are weak, of −3.51 and −6.27 kJ/mol in tClAB and tBrAB, respectively. 

The magnitude of the strength of all these interactions smaller than −13.39 kJ/mol (−9.37 kJ/mol after scaling according to Afonin and Kuznetsov) suggests that they are weak and dominated by dispersion, which is in good agreement with the results obtained from CLP, Pixel or ESP analysis. The “overestimation” of energies of the intramolecular bonds results from the spatial crowding of neighbouring iodine atoms, whose electron clouds tightly overlap, which results in strong repulsion (due to steric hinderance of side-by-side atoms) and high V_BCP_, but also very strong dispersion due to London forces between pairs of neighbouring iodine atoms and high G_BCP_. This conclusion is in good agreement with the great contributions of the repulsion and dispersion terms to the total lattice energy.

A comparison of total interaction patterns in crystals using 3D HS, 2D FP and QTAIM in a set of congeners revealed that in adamantylated 4,5,6,7-tetrahalogeno-1*H*-benzimidazoles the distributions of interactions are determined to a greater degree by ethyl-adamantyl moiety than 4,5,6,7-tetrahalogeno-1*H*-benzimidazoles ring, while the number of interactions of a certain strength is to a greater degree modified by 4,5,6,7-tetrahalogeno-1*H*-benzimidazole ring than ethyl-adamantyl moiety. Overall, of the newly synthesized compounds, tIAB seems to be the most susceptible to the formation of a wide variety of intermolecular interactions, which are responsible for the ligand “fitting” and binding to the active site.

### 2.3. Chemical Reactivity

The chemical reactivity descriptors, Table 9, deliver some insight into the reactivity of the newly synthesized compounds.

**Figure 9:**
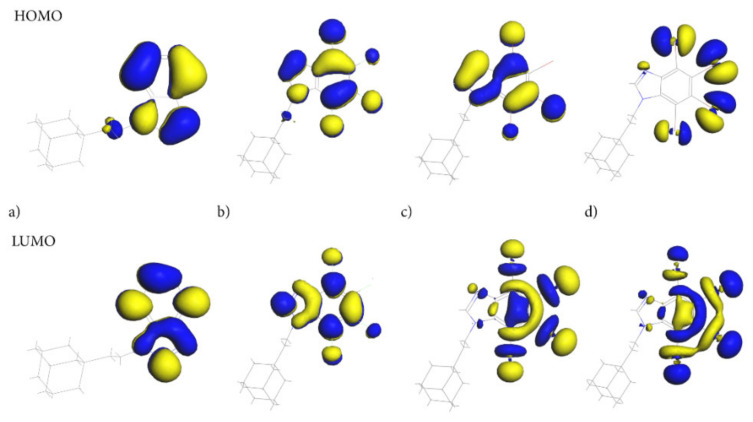
The Highest Occupied Molecular Orbital (HOMO) and the Lowest Unoccupied Molecular Orbital (LUMO) for adamantylated 4,5,6,7-tetrahalogeno-1*H*-benzimidazoles (**a**) AB, (**b**) tClAB, (**c**) tBrAB and (**d**) tBrAB. The blue represents the positive phase of the wave function and yellow represents the negative phase of the wave function.

Of particular importance are the frontier molecular orbitals: the Highest Occupied Molecular Orbital (HOMO) and the Lowest Unoccupied Molecular Orbital (LUMO). As the molecules of the compounds studied are uncharged, the HOMO/LUMO spatial distribution provides relevant information about the reactivity/stability of the specific regions of these molecules. Both HOMO and LUMO, Figure 9, are distributed exclusively over the 4,5,6,7-tetrahalogeno-1*H*-benzimidazole ring, indicating that this moiety should play a crucial role in biological activity. 

The ethyl-adamantyl moiety contributes neither to the HOMO nor the LUMO, which confirms that 4,5,6,7-tetrahalogeno-1*H*-benzimidazole ring depletes its charge. Detailed inspection of the HOMO and LUMO orbitals indicates that the path of electron density transfer upon the excitation (from the ground to the first excited state) highly depends on the halogen type. The HOMO distribution changes significantly upon the change in halogen from the carbon and nitrogen atoms in the AB heterocyclic ring to all halogen atoms in tIAB.

The HOMO orbital in tClAB is mainly located on the 1*H*-benzimidazole ring and chlorine atoms substituted at positions 1, 4 and 3, while in tBrAB it is mainly located on the ring and bromine atoms 1, 3 and 4. In tIAB, the HOMO orbital is mostly located on the iodine substituted at positions 2, 3, and 1, and to a lesser degree, 4 and N(7), i.e., the most reactive electrons are located at these positions. 

The LUMO delocalization site changes from the atoms in heterocyclic ring in AB to halogen atoms at positions 2, 3, and 4 of the heterocyclic ring (Figure 1) in tIAB. In tClAB, the LUMO orbital is distributed on the 4,5,6,7-tetrahalogeno-1*H*-benzimidazole ring and on chlorines 1, 4 and 3, while in tBrAB and tIAB, it is oriented toward the halogen atoms 2, 3 and 4 and, to a lesser degree, 1.

Therefore, intermolecular interactions should primarily involve halogen atoms in positions 2 and 3. An unusually low lying LUMO for tIAB suggests its easy participation in molecular reactions with nucleophiles (i.e., enhancement of accepting properties), Table 8. Conversely, a low-lying HOMO level for tClAB and tIAB suggests easy participation in molecular reactions with electrophiles. The HOMO-LUMO gap (i.e., the larger absolute hardness and smaller softness), higher for AB and tClAB than tIAB and tBrAB, refers to the higher kinetic stability and lower chemical reactivity of the former, because it is energetically unfavorable to add an electron to the high-lying LUMO in order to remove electrons from the low-lying HOMO. The smallest HOMO-LUMO gap (i.e., the smallest hardness) enhances the charge mobility. Thus, of four congener molecules, tIAB should be the softest, the least stable, the most reactive and the most easily excited photo-chemically. 

The ordering of the congeners studied according to the decreasing absolute electronegativity, χ, (a measure of the molecular ability to attract electrons to itself), is well correlated with that according to global packing (1), but not with that of halogen’s electronegativity (I < Br < Cl). Small, but positive, values of the maximum amount of electronic charge, ΔN_max_, suggests that molecules of the considered compounds act as electron acceptors. The most important descriptor, which measures the propensity of a species to accept the maximal number of electrons from a neighbouring reservoir of electron pool, is the electrophilicity index, ω, (literally the electrophilic power). It encompasses the tendency of an electrophile to acquire an extra amount of electron density, given by global chemical potential μ, and the resistance of a molecule to exchange electron density with the environment, given by global hardness η. Electrophilicity index of adamantylated 4,5,6,7-tetrahalogeno-1*H*-benzimidazoles takes values within the range 0.76–1.26, much lower than those for 4,5,6,7-tetrahalogeno-1*H*-benzimidazoles [66]. Thus, adamantylated 4,5,6,7-tetrahalogeno-1*H*-benzimidazoles are much weaker electrophiles than 4,5,6,7-tetrahalogeno-1*H*-benzimidazoles. Chemical substitution with four halogen atoms (Cl, Br or I), generating the electron-withdrawing inductive effect and simultaneously the electron-donating by resonance effect, leads to an increase in the electrophilicity index (ω = 0.83, 0.87, 0.76, 1.26 eV, for H, Cl, Br and I respectively). This effect is in contradiction to the decrease observed in 4,5,6,7-tetrahalogeno-1*H*-benzimidazoles (ω = 1.823, 1.765, 1.667 eV, for Cl, Br and I, respectively) [66], which are classified as strong electrophiles, and can be linked to electron-donating ethyl-adamantyl moiety. 

While tIAB, tClAB and AB are moderate electrophiles (0.8 < ω < 1.5), tBrAB is a weak electrophile (ω < 0.8). The best electrophile is tIAB, characterized by a high ω due to high |μ| value and a low η value. The electron-accepting power, ω_+,_ which is a measure of the propensity of a given system to accept electron density, is the highest for tIAB, followed by tClAB, while the electron-donating power, ω_-,_ which is the propensity of this system to donate electron density, is the lowest for tIAB, followed by tBrAB. The net electrophilicity, Δω _=_ ω_+_ + ω_−,_ i.e., electron-accepting power relative to its electron-donating power, is the highest for tIAB, followed by tClAB. Moreover, the tIAB molecule has the lowest value of global hardness, i.e., the lowest resistance towards the deformation or polarization of the electron cloud of the atoms. Thus, when the electrophilic activation, easy participation in molecular reactions with nucleophiles and global hardness are the key factors determining biological activity, tIAB should be as medicinally important a compound as 4,5,6,7-tetraiodo-1*H*-benzimidazole; however, when a high polarity of the molecule is of key importance, tBrAB would be more convenient, but when easier participation in molecular reactions with electrophiles is required, then tClAB will be preferred. Therefore, depending on the preferences of the particular protein, the ligand should show stronger biological activity.

### 2.4. Molecular Docking

Four ligands: AB and tClAB, tBrAB, tIAB were docked into rigid protein structures retrieved from the Protein Data Bank (PDB).

#### 2.4.1. Human Casein Kinase 2 (CK2)

X-ray based structure of the CK2 in the bound state with 4,5,6,7-tetrahalogeno-1H-benzimidazole (3KXN [67]) was retrieved from the PDB database. The procedure used for docking the ligands in the CK2 pocket was identical to that described earlier [66]. The potential binding site (cavity) of 308.375 Å^3^ in volume was detected. The cavity is formed by hydrophobic Ile66, Met163, Val45, Ile174 and Val116 and prefers a ligand that is nonpolar and has a specific shape ensuring the largest possible area of contact between the surfaces of the ligand and the receptor. 

**Figure 10:**
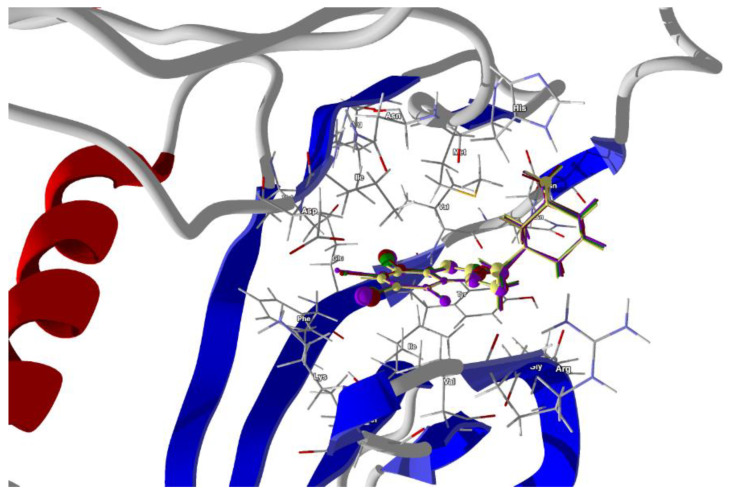
The docked poses of compound AB (yellow), tClAB (green), tBrAB (burgundy) and tIAB (purple) in a binding site of Human Casein Kinase 2, CK2. The protein backbone is represented as a cartoon, the binding cavity residues are shown as sticks and docked ligands are shown as a ball and a stick (the size of the ball is proportional to the strength of the interactions).

The search space of the simulation exploited in the docking studies was defined as a subset region of 9.0–15.0 Å around the active site cleft. After docking, the best poses that led to the stabilization of the complex with the highest binding/docking score were selected. The highest scoring ligands are shown in Figure 10. The ligands’ ordering according to the score is as follows:

tIAB > tBrAB > tClAB > AB >> actual ligand TIBI(4)

The best scores are for tIAB. The van der Waals interactions are the strongest for tIAB (−26.24 kJ/mol), followed by tBrAB (−26.16 kJ/mol), tClAB (−25.66 kJ/mol) and AB (−25.56 kJ/mol), analogously to the crystalline structure.

The steric contributions to the total protein–ligand binding, stronger than van der Waals, are the largest for tBrAB, followed by tIAB, tClAB and AB and almost twice as large as those for the actual TIBI ligand, Table 10.

The total protein–ligand binding energy of AB, tClAB, tBrAB and tIAB is greater than for the actual TIBI ligand. The ligands’ ordering according to the total protein–ligand binding energy is the same as (4). The scores and magnitude of total protein–ligand binding energy point to tIAB as the best ligand. Thus, the most effective filling of the hydrophobic cavity is achieved for this, possibly the largest and least polar ligand.

Despite the differences in the strength of binding, the protein–ligand binding modes do not differ significantly. The ligand tIAB binds with Ile174 (−11.788 kJ/mol), Met163 (−11.59 kJ/mol), Val53 (−9.96 kJ/mol), Val45 (−8.39 kJ/mol), His160 (−8.79 kJ/mol), Asp120 (−7.46 kJ/mol), Ile66 (−7.39 kJ/mol), Gly46 (−5.42 kJ/mol) and Phe113 (−4.94 kJ/mol). The ligand tBrAB binds with Ile174 (−11.96 kJ/mol), Met163 (−11.64 kJ/mol), Val53 (−9.69 kJ/mol), Val45 (−8.73 kJ/mol), His160 (−7.60 kJ/mol), Asp120 (−7.48 kJ/mol), Ile66 (−7.41 kJ/mol), Gly46 (−5.35 kJ/mol) and Phe113 (−4.82 kJ/mol). The ligand tClAB binds with Ile174 (−12.22 kJ/mol), Met163 (−11.37 kJ/mol), Val53 (−10.16 kJ/mol), Val45 (−8.33 kJ/mol), His160 (−8.12 kJ/mol), Asp120 (−7.57 kJ/mol), Ile66 (−7.44 kJ/mol), Gly46 (−4.90 kJ/mol) and Phe113 (−4.80 kJ/mol). The ligand AB binds with Met163 (−10.08 kJ/mol), Val53 (−9.14 kJ/mol), His160 (−8.21 kJ/mol), Val45 (−7.84 kJ/mol), Asp120 (−7.57 kJ/mol), Ile174 (−7.30 kJ/mol), Ile66 (−5.50 kJ/mol), Gly46 (−4.10 kJ/mol) and Asn118 (−3.91 kJ/mol). Although the interaction pattern is the same, the type of the halogen atom strongly determines the strength of the binding with the target residuum. The volume of the ligands will be an obstacle that can be avoided as long as the elasticity of the whole pocket is large enough. In the halogen-free AB, the strongest binding elements are carbon atoms C(2) and ethyl moiety, while in tClAB, tBrAB and tIAB, the strongest binding elements are halogen atoms X(4), X(3) and X(2) substituted at 4, 3 and 2 position of the ring and carbon C(2). X(2) linking Ile174, X(4) linking Val53, C(6) linking Val45 and adamantyl carbon linking Met163 seem to be the key factors for the formation of a strong binding. 

The stronger binding of all the congeners studied with CK2, i.e., the more favorable ligand-protein energy than that of the actual TIBI ligand at the binding cavity of CK2, suggests that the newly synthesized congeners show competitive inhibition with TIBI, and thus may be pharmaceutically attractive. The most promising seem to be tIAB.

#### 2.4.2. Membrane Matrix 2 Protein (M2)

**Figure 11:**
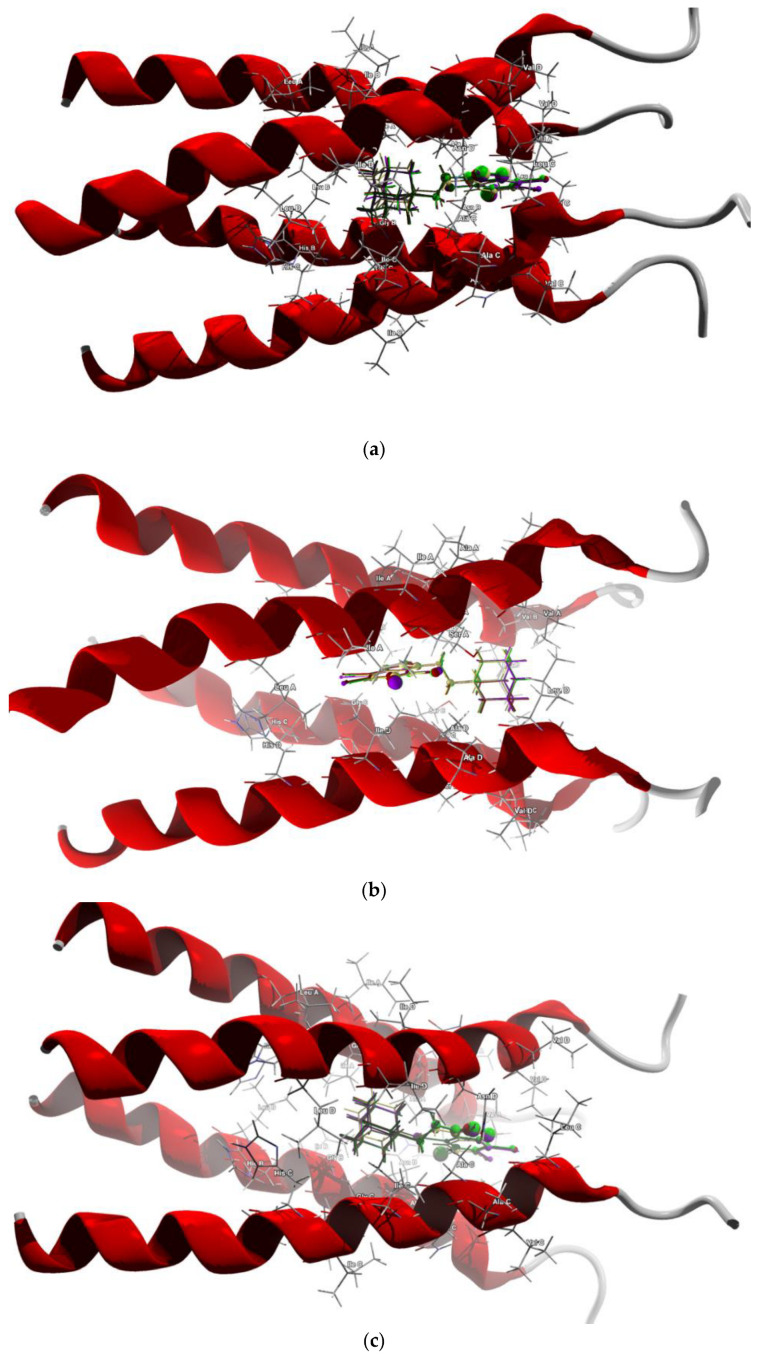
The docked poses of compound AB (yellow), tClAB (green), tBrAB (burgundy) and tIAB (purple) in binding site of Membrane Protein, M2: (**a**) 2LY0, (**b**) 2MUW, (**c**) 2MUV. The protein backbone is represented as a cartoon, the binding cavity residues are shown as sticks and docked ligands are shown as ball and stick (the size of the ball is proportional to the strength of the interactions).

The membrane matrix 2 protein M2 of influenza A virus assembles as a tetrameric proton-conducting channel and a single molecule of the drug binds with amino acids forming the cavity. The PDB database offers only five structures of influenza A virus bound with the ligand, from among which we selected three ones for docking. Solution NMR-based structure of the influenza A virus S31N mutant in the bound state with (3s,5s,7s)-N-{[5-(thiophen-2-yl)-1,2-oxazol-3-yl]methyl}tricyclo[3.3.1.1~3.7~]decan-1-aminium (2LY0 [68]) and Nuclear Overhauser Effect (NOE) based model of the influenza A virus S31N mutant bounded with (3s,5s,7s)-N-[(5-bromothiophen-2-yl)methyl] tricyclo[3.3.1.1~3.7~]decan-1-aminium (2MUW and 2MUV [69]) were retrieved from the Protein Data Bank. Both M2 structures are in the states that represent the pharmacological target. The procedure used for docking the ligands in the proton channel was similar to that described above. PDB structures often require corrections due to the poor or missing assignments of hydrogen atoms of the receptor, bond orders, hybridization or charges; therefore, the necessary preparations were made. The potential binding sites (cavities) of 384 Å^3^ in volume in 2LY0, of 466 Å^3^ in 2MUV and of 741 Å^3^ in 2MUW were detected. The cavity in 2LY0 is mainly formed by the hydrophobic Val27, Ala30, polar Asn31, and Gly34 (hard to assign to any class). In 2MUV, the binding site is formed by Ala30, Val27, Asn31, Gly34 and additionally by hydrophobic Leu26, while in 2MUW, by hydrophobic Val27, strongly hydrophobic Ile33 and Ile26, Gly34 and polar Ser31. Different techniques, template docking and docking with defined searched space around the active site, both with rigid and flexible conformation of ligand and flexible sidechains, were probed. The template for docking was constructed using both known ligands, i.e., (3s,5s,7s)-N-{[5-(thiophen-2-yl)-1,2-oxazol-3-yl]methyl}tricycle[3.3.1.1~3.7~]decan-1-aminium and (3s,5s,7s)-N-[(5-bromo-thiophen-2-yl)methyl]tricycle[3.3.1.1~3.7~]decan-1-aminium. The search space of the simulation exploited in the docking studies was defined as a subset region of 9.0–15.0 Å around the active site cleft. Because of the high flexibility of the ligand (three torsions), conformational positions for each ligand were searched for. Although the ligand docking in the proton channel was successful for two orientations (i.e., for 2LY0 and 2MUV the opposite to that for 2MUW), the strongest protein–ligand bonding was achieved for the orientation in which, similarly as in solid state, the electrostatic complementarity rule is maintained, which means that ethyl-adamantyl is immersed in the negative potential in the protein cavity, while 4,5,6,7-tetrahalogeno-1*H*-benzimidazole moiety is immersed in the positive potential. The best poses which led to the stabilization of the complex with the highest binding/docking score were selected. According to the score, the highest binding potential should have tClAB and the lowest—AB, Table 10. The 3D structures of the best scoring dockings are shown in Figure 11a–c.

According to the total energy of binding with protein, the newly synthesized ligands can be ordered as follows:

tClAB > tIAB > tBrAB > AB (docking with M2 from 2LY01) tClAB > tBrAB > tIAB > AB (docking with M2 from 2MUV) tClAB > tIAB > tBrAB > AB (docking with M2 from 2MUW)(5)

For the first two, nearly identical orderings, the ligand’s orientation with respect to the channel is the same, so the position in the cavity and local polarity of the ligand seem of importance. Variant b) of ordering (5) is in agreement with the ordering (2) according to ∠C(2)N(1)C(10)C(11) dihedral angle in solid phase, which is not surprising because the conformations of particular ligands after docking do not differ much from those in solid phase, despite the presence of -CH_2_CH_2_ as a flexible separator.

The docking studies suggest that the ligands did not form any hydrogen bonds with M2. The protein–ligand van der Waals interactions are also the strongest for tClAB (only in 2MUV case are strongest for tIAB) and the weakest for AB. The steric contributions to the total protein–ligand binding show the same trend but are much stronger than those of van der Waals, which is an important factor for binding. All this suggests that tClAB, despite moderate electrophilicity index, should be the best ligand for inhibiting M2 protein.

The target amino acid residues are slightly different in the channels in 2LY01, 2MUV and 2MUW.

In 2LY01 channel, the target residues for the ligand AB are: Val27A (−11.98 kJ/mol), Val27B (−8.88 kJ/mol), Ala30A (−6.92 kJ/mol), Val27A (−6.70 kJ/mol), Ala30B (−6.54 kJ/mol), Ala30C (−5.24 kJ/mol), Ala30D (−4.66 kJ/mol) and Val27C (−4.56 kJ/mol). The target residues for the ligand tIAB are: Val27B (−9.52 kJ/mol), Val27A (−9.19 kJ/mol), Ala30B (−7.28 kJ/mol), Ala30A (−6.86 kJ/mol), Val27D (−6.57 kJ/mol), Ala30C (−5.96 kJ/mol), Ala30D (−5.52 kJ/mol) and Val27C (−4.19 kJ/mol).The target residues for the ligand tBrAB are: Val27B (−10.13 kJ/mol), Val27A (−9.20 kJ/mol), Ala30B (−7.48 kJ/mol), Ala30B (−7.48 kJ/mol), Val27D (−7.10 kJ/mol), Ala30A (−6.83 kJ/mol), Ala30C (−5.55 kJ/mol), Ala30D (−5.46 kJ/mol), Asn31B (−4.57 kJ/mol), Val27C (−4.34 kJ/mol). The target residues for the ligand tClAB are: Val27B (−9.62 kJ/mol), Val27B (−8.98 kJ/mol), Ala30A (−6.92 kJ/mol), Val27D (−6.61 kJ/mol), Ala30B (−6.38 kJ/mol), Ala30C (−5.82 kJ/mol), Ala30D (−5.59 kJ/mol) and Val27C (−5.13 kJ/mol). In AB, the strongest binding elements are carbon C(6) and nitrogen N(7), while in tClAB, tBrAB and tIAB, the strongest binding elements are carbon C(6), halogen atom X(4) substituted at 4 position of the ring and nitrogen N(7).

In 2MUV channel, the ligand tIAB binds with Ala30D (−8.46 kJ/mol), Val27A (−8.38 kJ/mol), Ala30C (−8.06 kJ/mol), Val27D (−7.53 kJ/mol), Ala30B (−6.53 kJ/mol), Asn30D (−6.52 kJ/mol), Val27C (−6.15 kJ/mol), Ala30A (−4.40 kJ/mol) and Asn31B (−3.52 kJ/mol). The ligand tBrAB binds with Val27A (−9.46 kJ/mol), Ala30D (−8.60 kJ/mol), Val27C (−8.48 kJ/mol), Ala30C (−7.69 kJ/mol), Val27D (−7.62 kJ/mol), Ala30B (−6.51 kJ/mol), Asn30D (−6.50 kJ/mol), Ala30A (−4.63 kJ/mol) and Asn31B (−3.67 kJ/mol). The ligand tClAB binds with Val27C (−9.83 kJ/mol), Ala30D (−9.01 kJ/mol), Val27A (−8.98 kJ/mol), Ala30C (−8.34 kJ/mol), Val27D (−8.21 kJ/mol), Asn30D (−6.95 kJ/mol), Ala30B (−6.42 kJ/mol), Ala30A (−4.79 kJ/mol) and Val27B (−3.76 kJ/mol). The ligand AB binds with Val27C (−8.99 kJ/mol), Ala30D (−8.31 kJ/mol), Ala30C (−7.11 kJ/mol), Val27D (−6.54 kJ/mol), Asn31D (−6.07 kJ/mol), Val27B (−5.90 kJ/mol), Ala30B (−4.55 kJ/mol), Val27A (−4.05 kJ/mol) and Ala30A (−3.34 kJ/mol).

Although the target amino acid residues in 2MUV and 2LY01 for all four ligands are the same, the type of the halogen atom strongly determines the strength of the binding. In halogen-free AB, the strongest binding elements are carbon atoms C(6) and both nitrogen atoms N(5) and N(7), while in tClAB, tBrAB and tIAB, the strongest binding elements are halogen atom X(3) substituted at position 3 of the ring, carbon C(6) and both nitrogen atoms N(5) and N(7).

In 2MUW channel, the ligand tIAB binds strongly with Ala30A (−9.18 kJ/mol), Ala30B (−8.65 kJ/mol), Ala30D (−8.54 kJ/mol), Ser 31A (−4.49 kJ/mol) Val27A (−4.34 kJ/mol), Val27D (−4.22 kJ/mol) and Ala30C(−4.15 kJ/mol). The ligand tBrAB binds strongly with Ala30A (−10.26 kJ/mol), Ala30D (−8.64 kJ/mol), Ala30B (−8.59 kJ/mol), Ser 31A (−4.45 kJ/mol), Val27D (−4.40 kJ/mol), Val27A(−4.09 kJ/mol), Ile33D (−3.97 kJ/mol) and Ala30C (−3.73 kJ/mol). The ligand tClAB binds strongly with Ala30A (−10.75 kJ/mol), Ala30D (−9.00 kJ/mol), Ala30B (−8.65 kJ/mol), Ile33D (−4.76 kJ/mol), Ser 31A (−4.29 kJ/mol), Val27D (−4.24 kJ/mol), Val27A (−4.17 kJ/mol), and Ala30C (−3.41 kJ/mol). The ligand AB binds strongly with Ala30A (−9.55 kJ/mol), Ala30B (−8.09 kJ/mol), Ala30D (−6.76 kJ/mol), Val27D (−4.18 kJ/mol), Val27A (−4.06 kJ/mol), Gly34A (−3.84 J/mol) and Ala30C (−3.15 kJ/mol).

In AB, the strongest binding elements are carbon atom C(6) and both nitrogen atoms N(5) and N(7), while in tClAB, tBrAB and tIAB, the strongest binding elements are halogen atom X(3) substituted at position 3 of the ring, carbons C(3), C(6), H(4) substituted at position 4 of the ring and both nitrogen atoms N(5) and N(7).

Irrespective of the source of M2 channel (2MUW, 2MUV or 2LY01), carbon C(6), nitrogen N(7) and halogen atoms X(3) or X(4) of the newly synthesized ligands and their binding with non-polar hydrophobic amino acids Ala30 and Val27 are the key factors for the M2 channel blocking. Thus, in each case, 4,5,6,7-tetrahalogeno-1*H*-benzimidazole moiety seems to play a more important role in the protein–ligand binding than ethyl-adamantyl. However, electrostatic complementarity (provided by ethyl-adamantyl) is important because it determines the orientation of the ligands in the M2 channel.

#### 2.4.3. SARS-CoV-2 Spike Glycoprotein (S Protein)

The cryo-electron microscopy structure of the surface spike glycoprotein (S protein) of SARS-CoV-2 (6M17 [70]) was retrieved from the PDB database. The procedure used for docking the ligands in the pocket was identical to that described above. The potential binding sites, adjacent binding sites, of the volumes of 274.536 Å^3^ and 209.520 Å^3^ were detected. The smaller cavity is formed by hydrophobic Phe277, Gly51, Val50 and polar, but not charged, Tyr129, Phe48, Ser280, Ser431, Phe283, Asn435, Ser278, Cys49. The larger cavity is formed by hydrophobic Gly395, Trp56 and polar, but not charged, Ser487 Tyr60, Ser483, Asp486, Asp270, Thr496 and hydrophilic Arg57. Taking into account the volumes of the cavity and the ligands, it was not certain that the docking of the newly synthesized ligands in a small cavity would succeed, but the scores obtained upon docking seemed promising. After docking, the best poses which led to the stabilization of the complex with the highest binding/docking score were selected. The highest scores were obtained for tIAB, followed by AB. The total interactions and steric energies of AB, tClAB, tBrAB and tIAB are much lower than those of the actual ligands: Amantadine or Rimantadine, Table 10. Taking into account the total energy of protein–ligand binding, the best seems to be AB, but the van der Waals repulsion is unfavorable. At sufficient proximity, van der Waals forces should stabilize the attraction. A detailed analysis reveals that van der Waals forces are repulsive for each new ligand, which is mainly a result of a too close proximity of halogen atoms (substituted at 1, 2 and 3 positions of the ring) to amino acids (i.e., too large size of the ligand). The magnitude of van der Waals forces points to tIAB as the best ligand, although the repulsion caused by halogens is still an obstacle.

**Figure 12:**
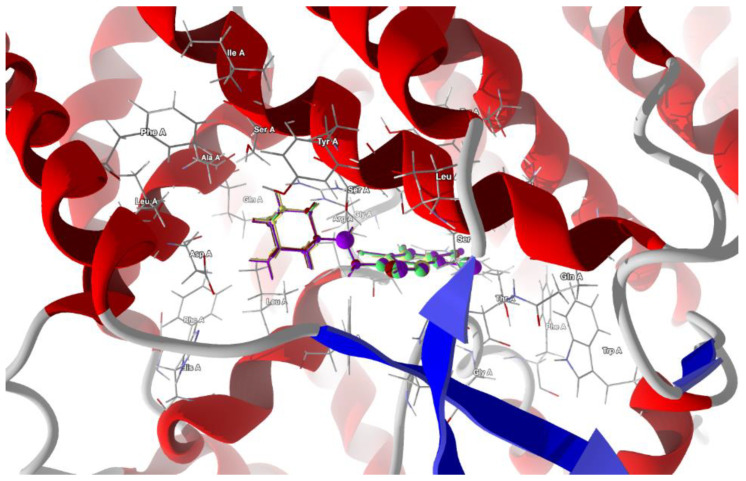
The docked poses of compound AB (yellow), tClAB (green), tBrAB (burgundy) and tIAB (purple) in binding site of Severe Acute Respiratory Syndrome Coronavirus 2, SARS-CoV-2 (6M17). The protein backbone is represented as a cartoon, the binding cavity residues are shown as sticks and docked ligands are shown as ball and stick (the size of the ball is proportional to the strength of the interactions).

Docking in the larger cavity is more successful and leads to the stabilization of the complex with a higher binding/docking score than that of the actual ligands and with the desired van der Waals attraction instead of the repulsion. The best poses which led to the stabilization of the complex with the highest score were selected, Table 10. The 3D structures of best scoring dockings are shown in Figure 12.

The ligands’ ordering according to the scores and protein–ligand energy of binding is as follows:

tClAB ≈ tBrAB > tIAB > AB >> actual ligand Rimantadine >> actual ligand Amantadine (6)

The total energy of protein–ligand binding is the highest for tBrAB. The van der Waals interactions are attractive and the strongest for AB (−28.93 kJ/mol), followed by those for tBrAB (−27.72 kJ/mol), tClAB (−27.69 kJ/mol) and tIAB (−25.86 kJ/mol), and all are of a similar order of magnitude as those of the actual ligands. The steric contribution to the total protein–ligand binding is the largest for tClAB and the smallest for AB, while repulsion is the largest for tIAB. The interaction pattern for each ligand is different.

The target residues for the ligand tIAB are: Ser487 (−15.43 kJ/mol), Arg57 (−13.68 kJ/mol), Tyr60 (−12.47 kJ/mol), Gly395 (−12.27 kJ/mol), Ser483 (−11.31 kJ/mol), Asp270 (−7.55 kJ/mol), Asp486 (−6.9 kJ/mol), Leu61 (−4.98 kJ/mol), Leu482 (−4.92 kJ/mol) and Ser491 (−4.52 kJ/mol). The target residues for the ligand tBrAB are: Ser487 (−15.76 kJ/mol), Arg57 (−15.46 kJ/mol), Tyr60 (−12.67 kJ/mol), Ser483 (−12.47 kJ/mol), Gly395 (−12.39 kJ/mol), Asp270 (−7.91 kJ/mol), Asp486 (−7.58 kJ/mol), Trp56 (−5.35 kJ/mol), Leu61 (−5.11 kJ/mol), Ser491 (−4.69 kJ/mol), Thr396 (−4.59 kJ/mol) and Leu482 (−4.25 kJ/mol). The target residues for the ligand tClAB are: Arg57 (−16.74 kJ/mol), Ser487 (−15.49 kJ/mol), Tyr60 (−12.79 kJ/mol), Gly395 (−12.55 kJ/mol), Ser483 (−12.26 kJ/mol), Asp486 (−8.39 kJ/mol), Asp270 (−7.89 kJ/mol), Trp56 (−6.43 kJ/mol), Thr396 (−5.59 kJ/mol), Leu61 (−5.28 kJ/mol), Ser491 (−4.59 kJ/mol) and Leu482 (−4.22 kJ/mol). The target residues for the ligand AB are: Arg57 (−17.42 kJ/mol), Ser487 (−15.41 kJ/mol), Tyr60 (−11.14 kJ/mol), Ser483 (−10.67 kJ/mol), Asp486 (−7.55 kJ/mol), Asp270 (−5.42 kJ/mol), Leu61 (−5.42 kJ/mol), Ser491 (−4.76 kJ/mol) and Thr396 (−4.57 kJ/mol).

In AB, the strongest binding elements are the carbon of ethyl moiety, C(6) and nitrogen N(7), while in tIAB, the strongest binding elements are the carbons of ethyl moiety and C(6), halogen atom X(1) substituted at position 1 of the ring and nitrogen N(7). However, in tClAB and tBrAB, the strongest binding elements are halogen atom X(1) substituted at 1 position of the ring and carbons C(6), ethyl moiety and nitrogen N(7). This clearly noticeable division is consistent with the isostructurality. The strong hydrogen bond, linking nitrogen N(7) (acceptor) and Ser 483 (donor), seems to be an additional key factor for the strong binding of the newly synthesized ligands with spike protein. Its length varies from 3.0293 Å for AB (of −2.5 kJ/mol), 2.9403 Å for tBrAB (of −2.5 kJ/mol), 2.9148 Å for tClAB (of −2.49 kJ/mol) and 2.8938 Å for tIAB (of −2.5 kJ/mol).

**Figure 13:**
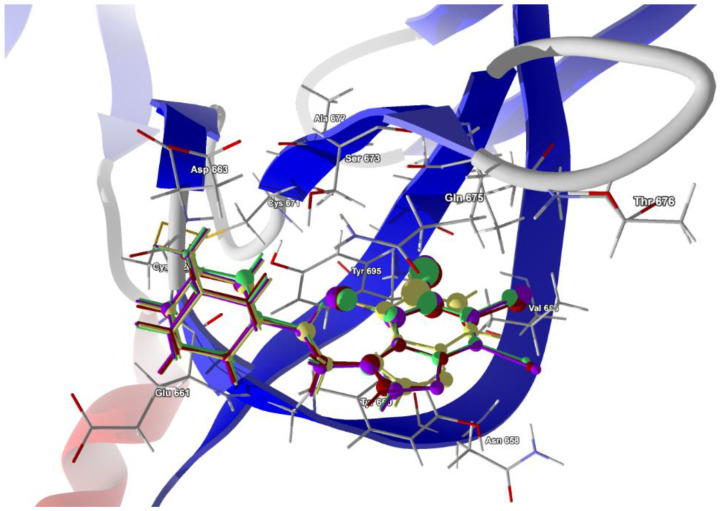
The docked poses of compound AB (yellow), tClAB (green), tBrAB (burgundy) and tIAB (purple) in binding site of Severe Acute Respiratory Syndrome Coronavirus 2, SARS-CoV-2 (7QUR). The protein backbone is represented as a cartoon, the binding cavity residues are shown as sticks and docked ligands are shown as a ball and stick (the size of the ball is proportional to the strength of the interactions).

The second structure, i.e., electron microscopy structure of the SARS-CoV-2 spike protein with ethylbenzamide-tri-iodo siallyllactose (7QUR [71]), was also retrieved from the PDB. The procedure used for docking the ligands in the pocket was similar to that described above, but we used only the template docking technique. The best poses which led to the stabilization of the complex with the highest binding/docking score were selected, Table 10. The 3D structures of the best scoring dockings are shown in Figure 13.

The conformation of ligands docked in the protein cavity is slightly different from that in solid state. According to the score, the highest binding potential should have tBrAB, while the lowest, AB, but the differences between tBrAB and two other halogen-containing ligands (tIAB and tClAB) are very small. The protein–ligand van der Waals interactions are the strongest for AB and the weakest for tClAB. The steric contribution to the total protein–ligand binding, much stronger than van der Waals interactions, is the largest for tClAB and the smallest for AB. The strongest protein–ligand interactions in tBrAB and tClAB suggest that they should be the best ligands for the SARS-CoV-2 spike protein (similarly to the M2 channel blocking).

The ligand tIAB binds with Tyr660 (−34.66 kJ/mol), Gln675 (−24.59 kJ/mol), Glu661 (−13.90 kJ/mol), Asp663 (−10.09 kJ/mol), Ser673 (−8.35 kJ/mol), Tyr695 (−7.08 kJ/mol), Cys662 (−5.79), Ile693 (−4.37 kJ/mol) and Val656 (−2.31 kJ/mol). The ligand tBrAB binds with Tyr660 (−35.62 kJ/mol), Gln675 (−24.86 kJ/mol), Glu661 (−12.80 kJ/mol), Asp663 (−10.81 kJ/mol), Tyr695 (−8.98 kJ/mol), Ser673 (−8.21 kJ/mol), Cys662 (−5.95 kJ/mol), Ile693 (−5.63 kJ/mol) and Val656 (−2.37 kJ/mol). The ligand tClAB binds with Tyr660 (−35.73 kJ/mol), Gln675 (−25.78 kJ/mol), Glu661 (−13.89 kJ/mol), Tyr695 (−9.89 kJ/mol), Asp663 (−9.89 kJ/mol), Ser673 (−8.49 kJ/mol), Cys662 (−5.89 kJ/mol), Ile693(−5.99 kJ/mol) and Val656 (−2.16 kJ/mol). The ligand AB binds with Tyr660 (−30.17 kJ/mol), Gln675 (−21.49 kJ/mol), Glu661 (−23.67 kJ/mol), Tyr695 (−7.67 kJ/mol), Asp663 (−9.98 kJ/mol), Ser673 (−6.68 kJ/mol), Cys662 (−5.64 kJ/mol), Ile693 (−2.82 kJ/mol) and Val656 (−0.55 kJ/mol). The docking studies revealed that ligands did not form any hydrogen bonds with the residues.

In AB, the strongest binding elements are the carbon atoms C(3), C(2) and C(4), while in tClAB, tBrAB and tIAB, the strongest binding elements are the halogen atoms (Cl, Br or I) substituted at positions 3 and 4 of the ring and carbon atoms C(3), C(4). X(3) links to Ile693 and Tyr695, and X(4) links to Tyr660 and Tyr 695. Moreover, the Tyr660 and Tyr 695 residues participate in the π-π stacking interaction with benzimidazole ring. The halogens occupying positions 3 and 4 are very important for the formation of interactions, while this at position 1 is of no importance. This is in agreement with the conclusions derived from Hirshfeld surface analysis. The strong score and total binding energy of all the newly synthesized ligands with SARS-CoV-2 spike protein suggests that they can be pharmaceutically relevant. Moreover, the addition of ethyl-adamantyl moiety seems to broaden and modify the therapeutic indices of the 4,5,6,7-tetrahalogeno-1*H*-benzimidazoles.

## 3. Experimental Section 

^1^H NMR spectra were recorded on a Bruker Avance (300 MHz) spectrometer. Chemical shifts (δ) are quoted in parts per million (ppm) downfield of tetramethylsilane, using protonated solvent as internal standard (DMSO-d_6_ at 2.50 ppm).

### 3.1. General Procedure for the Synthesis and Single Crystal Growth

**1-[2-(Adamantyl)ethyl]-1*H*-benzimidazole (AB).** Benzimidazole (2 mmol, 236 mg) was dissolved in 15 cm^3^ of dry ethanol and 2.5 mmol of EtONa in dry ethanol was added. Into this mixture, 3 mmol (739 mg) of 1-(2-bromoethyl)adamantane were added and the solution was boiled for over 6 h. Then, ethanol was distilled off. The crude product was purified using column chromatography (CHCl_3_/MeOH; 95:5) method. M.p. 137 °C; yield: 71%. ^1^H NMR (DMSO) δ (ppm): 8.46 (s, 1H, H-C), 7.68 (m, 2H, H-C_ar._), 7.18 (m, 2H, H-C_ar._), 4.50 (m, 2H, CH_2_), 3,50–1.55 (m, 17H, CH_2_-Ad). Anal. Calcd for C_19_H_24_N_2_: C, 81.40; H, 8.60; N, 10.00. Found: C, 81.33; H, 8.49; N, 9.88. 

Single crystals used for X-ray data collection were grown by spontaneous evaporation of ethyl acetate:hexane solution of AB at room temperature. 

**1-[2-(Adamantyl)ethyl]-4,5,6,7-tetrachloro-1*H*-benzimidazole (tClAB).** A quantity of 4,5,6,7-Tetrachlorobenzimidazole (2 mmol, 512 mg) was dissolved in 15 cm^3^ of dry ethanol and 2.5 mmol of EtONa in ethanol was added. To this mixture, 3 mmol (739 mg) of 1-(2-bromoethyl)adamantane were added and the solution was boiled for over 6 h. Then, ethanol was distilled off. The crude product was purified using column chromatography (CHCl_3_/MeOH; 95:5) method. M.p. 183 °C; yield: 64%. ^1^H NMR (DMSO) δ (ppm): 8.56 (s, 1H, H-C), 4.55 (m, 2H, CH_2_), 3,20–1.55 (m, 17H, CH_2_-Ad). Anal. Calcd for C_19_H_20_Cl_4_N_2_: C, 54.61; H, 4.79; N, 6.72. Found: C, 54.33; H, 4.71; N, 6.80. 

Single crystals used for X-ray data collection were grown by spontaneous evaporation of ethyl acetate: hexane solution of tClAB at room temperature. 

**1-[2-(Adamantyl)ethyl]-4,5,6,7-tetrabromo-1*H*-benzimidazole (tBrAB).** A quantity of 4,5,6,7-Tetrabromobenzimidazole (2 mmol, 868 mg) was dissolved in 15 cm^3^ of dry ethanol and 2.5 mmol of EtONa in ethanol was added. Into this mixture, 3 mmol (739 mg) of 1-(2-bromoethyl)adamantane were added and the solution was boiled for over 10 h. Then, ethanol was distilled off. The crude product was purified using column chromatography (CHCl_3_/MeOH; 95:5) method. M.p. 205 °C; yield: 30%. ^1^H NMR (DMSO) δ (ppm): 8.23 (s, 1H, H-C), 4.54 (m, 2H, CH_2_), 3,10–1.55 (m, 17H, CH_2_-Ad). Anal. Calcd for C_19_H_20_Br_4_N_2_: C, 33.31; H, 3.40; N, 4.72. Found: C, 33.33; H, 3.41; N, 4.80. 

Single crystals used for X-ray data collection were grown by spontaneous evaporation of ethyl acetate: hexane solution of tBrAB at room temperature. 

**1-[2-(Adamantyl)ethyl]-4,5,6,7-tetraiodo-1*H*-benzimidazole (tIAB).** A quantity of 4,5,6,7-Tetraiodobenzimidazole (1 mmol, 621 mg) was dissolved in 15 cm^3^ of dry ethanol and 1.5 mmol of EtONa in ethanol was added. Into this mixture, 2 mmol (486 mg) of 1-(2-bromoethyl)adamantane were added and the solution was boiled for over 10 h. Then, ethanol was distilled off. The crude product was purified using column chromatography (CHCl_3_/MeOH; 95:5) method. M.p. 225 °C (decomp.); yield: 20%. ^1^H NMR (DMSO) δ (ppm): 7,90 (s, 1H, H-C), 4.54 (m, 2H, CH_2_), 3,00–1.55 (m, 17H, CH_2_-Ad). Anal. Calcd for C_19_H_20_I_4_N_2_: C, 29.12; H, 2.60; N, 3.63. Found: C, 29.39; H, 2.48; N, 3.70. 

Single crystals used for X-ray data collection were grown by spontaneous crystallization of **t**IAB with THF.

### 3.2. X-ray Single Crystal Studies and Structure Solution

X-ray structural studies of 1-[2-(1-adamantyl)ethyl]-1*H*-benzimidazole (AB) and 1-[2-(1-adamantyl)ethyl]-4,5,6,7-tetrahalogeno-1*H*-benzimidazoles ((X=Cl, Br or I; tClAB, tBrAB and tIAB) were performed at room temperature (RT) using an Xcalibur R single crystal diffractometer from Oxford Diffraction. Monochromated CuKα radiation was applied. Monocrystals of the studied compounds were mounted on the goniometer and reflections were collected up to a Bragg angle 2θ ≤ 140°. The intensities of the reflections were corrected for Lorenz-polarization effects and for absorption and extinction. The details of the experimental procedure are listed in Table 1. The structures were solved using direct methods from SHELXS-98 program [72] and then refined by application of SHELXL-98 software [28]. All non-hydrogen atoms were refined anisotropically, whereas the positions of all hydrogen atoms were calculated and their thermal parameters were refined isotropically. Crystallographic information files (CIF) of AB, tClAB, tBrAB and TIAB have been deposited with Cambridge Structural Database Centre (CCDC 1478829, CCDC 1478831, CCDC 1478832, CCDC 1478834).

### 3.3. CLP/PIXEL

Two theoretical approaches, CLP [60] and Pixel [61] have been used to evaluate intermolecular potential energies. In the CLP (atom-atom) approach, potentials depend only on the distance between atomic nuclei and are purely empirical. Evaluation of the crystal lattice energy requires only knowledge of the cell dimensions and atomic coordinates. The PIXEL approach is more sophisticated. Intermolecular energies are calculated as numerical integrals over a large number of small volumes (electron density units), which are called “pixels”. The method requires ab initio calculations to obtain a grid of discrete values of electron density values. The individual contributions to the total energy: Coulombic (electrostatic), polarization, dispersion and Pauli repulsion are then calculated between pairs of pixels and nuclei in different molecules. Their sum is equal to the lattice energy. 

### 3.4. DFT/QTAIM/RDS/Hirshfeld Surfaces

Quantum chemical calculations required for QTAIM analysis were carried out within the Density Functional Theory (DFT) approach rooted in the Kohn–Sham [73] theorem generalized by Levy [74]. For the bulk solid, the calculations of B3LYP scheme (functional of Becke B88 [75] combined with the Lee–Yang–Parr correlation functional LYP [76]) with Grimme [77] correction on dispersion (D3) and numerical radial functions basis triple-zeta were applied. All calculations were performed using Gaussian 09 rev. D01 [78]. The X-ray crystallography at usual resolution often fails to directly access the positions of light atoms, mainly because they show weak contribution to the diffraction; as a consequence, the proton positions are poorly identified. Therefore, the corrected (optimized) proton positions are routinely used by us in any analyses based on the electron density distribution [62,63,64,65,66].

Theoretical analysis of the topology of intermolecular interactions was performed within the Bader’s Quantum Theory of Atoms in Molecules (QTAIM) [55] supplemented with the Reduced Density Gradient (RDS) [56] technique. The analysis of the stationary points (maxima, saddle points, or minima in the electron density) within QTAIM permits differentiation of the nucleus-, bond-, ring-, and cage-critical points, denoted as NAP (Nuclear Critical Point), BCP (Bond Critical Point), RCP (Ring Critical Point), and CCP (Cage Critical Point). The topological descriptors calculated at the BCPs, saddle points at which the gradient in the electron density, ρ(r), vanishes, deliver the information about the bonds’ strength and nature. The calculated quantities include the electron density at the BCP, ρ(r_BCP_), and three eigenvalues of principal components of Hessian matrix composed of second partial derivatives of the electron density which describe curvature of the electron density according to the principal axes perpendicular to the bond, λ_1_, λ_2_, and along the bond, λ_3_, the sum of Hessian eigenvalues (Laplacian), Δρ, a widely reported quantity to discern between bond types. The Laplacian summarizes three eigenvalues of different signs, which may lead to relatively large uncertainties. Therefore, Espinosa [79] introduced the curvature along the bond, λ_3_, as an alternative measure of bond strength. But the hydrogen bond energy, E_HB_, can be estimated using well-known formulas derived using partitioning of the electron density scheme [80].

(7) $$E_{\mathrm{HB}}\approx E_{\mathrm{HBE}}=0.5V\left(r_{\mathrm{BCP}}\right)$$

by Espinosa−Molins−Lecomte [81]

(8) $$E_{\mathrm{HB}}\approx E_{\mathrm{HBM}}=-0.429G\left(r_{\mathrm{BCP}}\right)$$

by Matta [82]

(9) $$E_{\mathrm{HB}}\approx E_{\mathrm{HBEM}}=-223.08\rho \left(r_{\mathrm{BCP}}\right)+0.7423$$

by Emamian (for neutral complexes) [83]

(10) $$E_{\mathrm{HB}}\approx E_{\mathrm{HBA}}=-\left(0.277\left|V\left(r_{\mathrm{BCP}}\right)\right|+0.45\right)=-(0.554\left|E_{\mathrm{HBE}}|+0.45\right)$$

by Afonin [84].

A comparative analysis by Vener [85] and Emamian [83] shows that the more popular Equation (7) systematically overestimates E_HB_, while the value given by Equation (8) is reasonable. A survey of various methods often used to estimate the energies of intramolecular hydrogen bonds has been performed [86].

We compared the results obtained from the abovementioned Formulae (7)–(10) for the estimation of E_HB_. The analysis of the interactions according to QTAIM must be performed attentively because the results can be easily overinterpreted, especially in terms of the number and nature of bonds. Some authors [87,88,89,90] point out that the mere presence of a bond path between a pair of atoms cannot be treated as evidence of attractive interactions between these atoms. Therefore, the set of quantum indicators characterizing the bonds must be carefully analyzed. Despite these valid criticisms, experimental binding strengths are often in line with the model’s predictions. The critical approach is referred to specific cases of intramolecular hydrogen bonds.

The homohalogen contacts energy can be estimated using Kuznetsov relationships [91]:

(11) $$E_{\mathrm{XX}}\approx E_{\mathrm{XXK}}=\{\begin{array}{c} -0.47G\left(r_{\mathrm{BCP}}\right)\;\mathrm{for}\;\mathrm{Cl}\\ -0.57G\left(r_{\mathrm{BCP}}\right)\;\mathrm{for}\;\mathrm{Br}\\ -0.67G\left(r_{\mathrm{BCP}}\right)\;\mathrm{for}\;I \end{array}$$

Exploration of intermolecular interaction pattern and packing capacities in solid was performed within the 3D Hirshfeld Surfaces (3D HS) approach [57,58]. Three-dimensional HS is the outer contour of the space, which a molecule occupies in a crystalline environment, but according to the Hirshfeld concept, the electron density of a molecule is divided into continuous atomic fragments. Three-dimensional HS was constructed with the use of the molecular weight-function (a quotient of promolecule and procrystal electron density). The descriptors like d_norm_, shape-index and curvedness of the surface mapped over 3D HS were evaluated [57]. The intermolecular interactions were visualized using red-white-blue scheme, where red was used for short contacts such as hydrogen bonds, white for contacts of about van der Waals radii, and blue for the remaining, much longer, contacts. The shape index and curvedness of the surface mapped over 3D HS described its flatness. The decomposition of the 3D HS into a 2D ‘molecular fingerprint’ (2D FP) map (plot of the distances of any surface point to the nearby interior and exterior atoms d*_i_* versus d*_e_*) [58], which summarizes the distribution of interactions of the molecule with its environment, was made. The surface contact data derived from the 2D FP were used to derive the enrichment ratio, E_XY_, a descriptor defined as the ratio between the proportion of the actual contacts in crystal and theoretical proportion of the random contacts [92], which indicate privileged and disfavored contacts between two atomic species, X and Y.

A further global quantitative characterization of the non-covalent interactions was delivered by the electrostatic potential (ESP) surface V_S_(r) (the molecular surface defined by electron density ρ(r) = 0.001 electron bohr^3^), mapped onto the 3D HS. Using this technique, a complete description of interactions based on the electrostatic complementarity within the so-called Politzer [59,93,94] GIPF (generalized interactions properties function) approach was possible. The parameters of ESP [95], i.e., the quantities providing its numerical characterization (*V_S_^+^, V_S_^−^,* their variances σ_+_^2^ and σ_−_^2^ and total variance σ_T_^2^, balance parameter (η), the average deviation from the mean surface potential (π)), were calculated.

The theoretical reactivity indices, the absolute electronegativity [χ = −(E_LUMO_ + E_HOMO_)/2; eV], chemical potential [μ = −χ; eV], absolute hardness [η = (E_LUMO_ − E_HOMO)_/2; eV)], electrophilicity index (reactivity) [ω = χ^2^/2η; eV] and softness [S = 1/η; 1/eV] were calculated using Par and Pearson HOMO (Highest Occupied Molecular Orbital)-LUMO (Lowest Unoccupied Molecular Orbital) approach [96]. The electron-accepting power [ω^+^; wV], electron-donating power, [ω^−^; eV] and net electrophilicity [Δω= ω^+^ + ω^−^; eV] were calculated using Gázquez definition [97]. For the studies of reactivity parameters, we applied MP2 functional, which is known to reproduce HOMO-LUMO correctly.

### 3.5. Molecular Docking

Molecular docking is a widely used technique in the screening of novel therapeutic agents [98], which requires not only the knowledge of the ligand structure but also reliable 3D X-ray crystal structure of protein. Both are crucial for accurate ligand docking. The conversion of the files with receptor and ligand structures to the .pdbqt format was made with MGLTools. Potential binding sites were identified using the cavity (pocket) detection algorithm CaverDock [99]. Molecular docking was performed using AutoDock [100] and AutoDock Vina [101]. Template docking and docking with defined searched space around the active site were probed. The best pose was selected on the basis of scoring function estimating the protein–ligand binding energy. The score being a linear combination of steric, van der Waals, hydrogen bonding, electrostatic terms, torsion and sp^2^-sp^2^ terms was used. In the next step, the residues (side chains) closest to active ligand were minimized with respect to the pose found and the ligand was energy-minimized using standard potentials. Finally, the total protein–ligand binding energy, steric and van der Waals contributions were calculated. The results of molecular docking were visualized using a Discovery Studio visualizer [102].

## 4. Conclusions

The newly synthesized compounds crystallize within two different space groups, P-1 (AB and tIAB) and P2_1_/c (tClAB and tBrAB). AB and tIAB are isostructural with 4,5,6,7-tetrafluoro-1*H*-benzimidazole, while tClAB and tBrAB are not isostructural with any of 4,5,6,7-tetrahalogeno-1*H*-benzimidazoles, 4,6-dibromo-5,7-diiodo-1*H*-benzimidazole, 4,6-dichloro-5,7-diiodo-1*H*-benzimidazole, 4,5,6,7-tetrahalogeno-phthalic anhydrides or adamantane.

Crystalline conformation of molecules of all four congeners is close and similar in the crystal and the protein pocket/channel. In halogen congeners, the conformation is stabilized by intramolecular C-H⋯Hal hydrogen bonds (two of −1.71 and −11.16 kJ/mol in tIAB.; three of −2.36, −5.25 and −8.01 kJ/mol in tBrAB.; −2.10, −4.99 and −8.27 kJ/mol in tClAB). In AB, the conformation is stabilized by C-H⋯H-C weak dispersive interactions of −9.79 kJ/mol. Strong, intramolecular Hal⋯Hal van der Waals contacts of −10.90 and −12.73 kJ/mol in tIAB, which result in the lack of flatness of 4,5,6,7-tetraido-1*H*-benzimidazole moiety, are absent in the other congeners studied. 

According to the total strength the intermolecular interactions in the crystalline lattice, the congeners can be ordered as follows: tIAB >> tClAB ≅ tBrAB >> AB and classified as dominated by dispersion. In AB, the strongest interactions, two C-H⋯N intermolecular hydrogen bonds of −9.20 kJ/mol, form a dimer. In tIAB, a dimer is formed by two C-H⋯N hydrogen bonds of −10.25 kJ/mol and two weak intermolecular C-H⋯Hal hydrogen bonds of −2.07 kJ/mol. In tClAB and tBrAB, dimers are formed with the use of a set of interactions (Hal⋯H-C, Hal⋯Hal, and Hal⋯π linking two 4,5,6,7-tetrahalogeno-1*H*-benzimidazole rings and three H⋯H contacts linking ethyl-adamantyl moieties). Hal⋯π are relatively strong (of −6 kJ/mol in tClAB and −6.73 kJ/mol in tBrAB), but the energies of the other (H⋯H, Hal⋯H-C, Hal⋯Hal contacts) do not exceed 3.2 kJ/mol. 

The difference in both scattering and number of particular intermolecular distributions in the congeners studied, strongly increase with increasing van der Waals radius of the halogen. This effect of scattering is about twice less pronounced than the change in intensity and the weakest for chlorine, but the strongest for iodine. A comparison of the interaction patterns in crystals in a set of congeners revealed that, in adamantylated 4,5,6,7-tetrahalogeno-1*H*-benzimidazoles, the distributions of interactions is determined to a greater degree by adamantyl than 4,5,6,7-tetrahalogeno-1*H*-benzimidazoles, while the number of interactions of a certain strength is to a greater degree modified by 4,5,6,7-tetrahalogeno-1*H*-benzimidazoles than adamantyl. In general, the 4,5,6,7-tetrahalogeno-1*H*-benzimidazole ring provides binding abilities and -CH_2_CH_2_ adds some flexibility, while the adamantyl moiety is responsible for the steric effects (steric hindrance and van der Waals repulsion).

The narrowest HOMO-LUMO gap and the highest electrophilicity from among all four congeners studied have been established for tIAB, which is the softest and the least stable (the smallest energy is required for its excitation). The magnitude of π, which describes the average deviation from the mean surface potential and local polarity of a molecule increases in the order AB < tClAB = tBrAB < tIAB. Thus, internal charge separation seems highly disturbed by the attachment of different halogen atoms. The, η_S_σ_T_^2^ correlates very well with the hydrogen-bond-accepting tendency and indicates weak hydrogen bonds in AB, but stronger ones in tIAB. 

The stronger binding of all the congeners studied with M2, CK2 and SARS-CoV-2 than those of the actual ligands, suggests that the newly synthesized congeners show competitive inhibition, and thus, are pharmaceutically attractive. tIAB seems more promising as a CK2 inhibitor and tClAB as an M2 blocker, while tBrAB or tClAB are more promising as SARS-CoV-2 inhibitors. Overall, of the newly synthesized compounds, tIAB seems to be the most susceptible to the formation of a wide variety of intermolecular interactions, while tClAB and tBrAB exert smaller repulsion. Pocket preferences for the number and type of bonds are responsible for the choice of the particular ligand that best “fits” and binds to the active site. The addition of ethyl-adamantyl moiety seems to broaden and modify the therapeutic indices of the 4,5,6,7-tetrahalogeno-1*H*-benzimidazoles.

## Figures and Tables

**Table 1:** Crystal data and structure refinement for AB, tClAB, tBrAB and tIAB.

	AB	tClAB	tBrAB	tIAB
No of deposit	CCDC 1478829	CCDC 1478831	CCDC 1478832	CCDC 1478834
Empirical formula	C_19_H_25_N_2_	C_19_H_20_Cl_4_N_2_	C_19_H_20_Br_4_N_2_	C_19_H_20_I_4_N_2_
Formula weight	281.41	418.17	596.01	783.97
Temperature	293(2) K	293(2) K	293(2) K	293(2) K
Wavelength	1.54184 Å	1.54184 Å	1.54184 Å	1.54184 Å
Crystal system	Triclinic	Monoclinic	Monoclinic	Triclinic
Space group	P-1	P 1 2_1_/c 1	P 1 2_1_/c 1	P-1
Unit cell dimensions	a = 6.9937(3) Å, γ = 70.000(3)°	a = 14.9004(4) Å	a = 15.1138(6) Å	a = 6.5333(2) Å, γ = 98.886(2)°
	b = 9.9348(4) Å, γ = 85.448(3)°	b = 12.0172(3) Å, γ = 98.071(3)°	b = 12.3261(7) Å, γ = 96.753(5)°	b = 10.9704(3) Å, γ = 98.514(2)°
	c = 12.0737(4) Å, γ = 85.551(3)°	c = 10.6991(3) Å	c = 10.7864(6) Å	c = 15.3814(3) Å, γ = 94.170(2)°
Volume	784.67(5) Å^3^	1896.81(9) Å^3^	1995.50(18) Å^3^	1072.03(5) Å^3^
Z	2	4	4	2
Density (calculated)	1.191 Mg/m^3^	1.464 Mg/m^3^	1.984 Mg/m^3^	2.429 Mg/m^3^
Absorption coefficient	0.528 mm^−1^	5.700 mm^−1^	9.876 mm^−1^	45.683 mm^−1^
F(000)	306	864	1152	720
Crystal size	0.3758 × 0.2469 × 0.1231 mm^3^	0.4219 × 0.3013 × 0.1671 mm^3^	0.2644 × 0.1388 × 0.0971 mm^3^	1.0284 × 0.0934 × 0.0185 mm^3^
Theta range for data collection	3.90 to 71.20°	3.00 to 71.09°	2.94 to 71.62°	2.95 to 71.19°
Index ranges	−8 ≤ h ≤ 8, −12 ≤ k ≤ 12, −14 ≤ l ≤ 14	−18 ≤ h ≤ 17, −14 ≤ k ≤ 14, −12 ≤ l ≤ 13	−18 ≤ h ≤ 18, −14 ≤ k ≤ 14, −13 ≤ l ≤ 12	−7 ≤ h ≤ 6, −13 ≤ k ≤ 13, −18 ≤ l ≤ 18
Reflections collected	22,919	25,427	16,160	30,943
Independent reflections	3029 [R(int) = 0.0380]	3654 [R(int) = 0.0323]	3802 [R(int) = 0.0806]	4136 [R(int) = 0.0464]
Completeness to theta = 71.20°	99.2%	99.6%	97.5%	99.6%
Absorption correction	Analytical	Analytical	Analytical	Analytical
Max. and min. transmission	0.941 and 0.853	0.505 and 0.197	0.471 and 0.200	0.469 and 0.008
Refinement method	Full-matrix least-squares on F^2^	Full-matrix least-squares on F^2^	Full-matrix least-squares on F^2^	Full-matrix least-squares on F^2^
Data/restraints/parameters	3029/0/193	3654/0/226	3802/0/226	4136/0/226
Goodness-of-fit on F^2^	1.040	1.046	1.791	1.101
Final R indices [I > 2sigma(I)]	R1 = 0.0434, wR2 = 0.1186	R1 = 0.0632, wR2 = 0.1876	R1 = 0.1300, wR2 = 0.4177	R1 = 0.0570, wR2 = 0.1746
R indices (all data)	R1 = 0.0524, wR2 = 0.1265	R1 = 0.0683, wR2 = 0.1934	R1 = 0.1397, wR2 = 0.4323	R1 = 0.0609, wR2 = 0.1780
Largest diff. peak and hole	0.174 and −0.226 e·Å^−3^	0.847 and −0.318 e·Å^−3^	3.881 and −1.041 e·Å^−3^	2.763 and −2.355 e·Å^−3^
Melting point	137 K	183 K	205 K	225 K (decomposition)

**Table 2:** Separation of coulombic/polarization/dispersion/repulsion terms to the total lattice calculated within CLP.

Molecular System	X	E_coulombic_ [kJ/mol]	E_polarization_ [kJ/mol]	E_dispersion_ [kJ/mol]	E_repulsion_ [kJ/mol]	E_total lattice_ [kJ/mol]
AB	H	−14.3	−29.1	−138.3	44.0	−137.6
tClAB	Cl	1.6	−23.5	−165.7	40.3	−147.3
tBrAB	Br	−5.9	−16.5	−191.5	55.0	−159.0
tIAB	I	−9.5	−15.1	−225.4	78.0	−172.0

**Table 3:** Separation of coulombic/polarization/dispersion/repulsion terms to the total lattice calculated within Pixel.

Molecular System	X	E_coulombic_ [kJ/mol]	E_polarization_ [kJ/mol]	E_dispersion_ [kJ/mol]	E_repulsion_ [kJ/mol]	E_total lattice_ [kJ/mol]	Dipole Moment
AB	H	−38.5	−16.9	−156.4	90.8	−121.0	4.522
tClAB	Cl	−30.4	−14.4	−189.5	76.0	−158.2	6.661
tBrAB	Br	−39.3	−16.4	−182.7	83.8	−154.7	7.724
tIAB	I	−75.4	−29.5	−224.8	162.2	−167.6	6.673

**Table 4:** Separation of coulombic/polarization/dispersion/repulsion terms to the total lattice calculated within Spackman scaled approach.

Molecular System	X	E_coulombic_ [kJ/mol]	E_polarization_ [kJ/mol]	E_dispersion_ [kJ/mol]	E_repulsion_ [kJ/mol]	E_total lattice_ [kJ/mol]	Level
AB	H	−29.1	−9.3	−130.0	63.3	−111.6	B3LYP
		−29.9	−15.6	−171.9	54.1	−152.7	HF
tClAB	Cl	−26.5	−9.1	−170.5	81.6	−133.3	B3LYP
		−20.8	−14.1	−182.4	62.9	−144.9	HF
tBrAB	Br	−42.1	−11.6	−157.6	86.4	−138.9	B3LYP
		−19.8	−21.2	−211.1	73.6	−175.4	HF
tIAB	I	-	-	-	-	-	B3LYP *
		−51.65	−15.8	−254.2	112.7	−201.7	HF

* basis set for Spackman’s scaled approach is unavailable.

**Table 5:** A comparison of % of the Hirshfeld surface, total Hirshfeld surface and volume characterizing a set of compounds studied.

Compound	X	X⋯X [%]	X⋯H [%]	H⋯X [%]	X⋯C [%]	C⋯X [%]	X⋯N [%]	N⋯X [%]	C⋯H [%]	H⋯C [%]	N⋯H [%]	H⋯N [%]	H⋯H [%]	N⋯N [%]	C⋯N [%]	N⋯C [%]	C⋯C [%]	Surface [Å^2^]	Volume [Å^3^]
AB	H	-	-	-	-	-	-	-	9.3	7.8	5.4	4.2	72.7	0.1	0.1	0.0	0.0	331.4	385.9
tClAB	Cl	1.0	25.3	17.4	3.8	3.9	1.2	1.1	5.1	4.0	3.8	3.3	29.1	0.0	0.2	0.2	0.6	383.2	467.2
tBrAB	Br	0.9	27.0	17.3	4.2	4.0	1.6	1.3	4.9	3.9	3.8	3.1	26.8	0.0	0.2	0.2	0.7	397.2	491.3
tIAB	I	8.6	22.4	12.6	5.4	5.1	0.5	0.4	3.5	2.8	3.3	2.9	29.9	0.3	0.5	0.4	0.8	423.9	526.9

**Table 6:** Enrichment ratios E_XY_ characterizing the various contacts in the set of compounds studied.

Molecular System	Atom	C	H	X	N
AB	Surface%	8.7	86.3	-	4.8
	C	0	-	-	-
	H	1.15	0.98	-	-
	N	0.12	1.12	-	0.42
tClAB	Surface%	9.2	58.6	27.3	4.8
	C	0.71	-	-	-
	H	0.85	0.85	-	-
	X	1.53	1.33	0.13	-
	N	0.34	1.26	0.84	0
tBrAB	Surface%	9.4	56.8	28.6	5.1
	C	0.79	-	-	-
	H	0.82	0.98	-	-
	X	1.53	1.36	0.11	-
	N	0.42	1.19	0.99	0
tIAB	Surface%	9.65	54	32.15	4.3
	C	0.86	-	-	-
	H	0.60	1.03	-	-
	X	1.69	1.03	0.83	-
	N	1.08	1.34	0.33	1.62

**Table 7:** Politzer-type statistical analysis of the ESP on the HSs: the minimum and maximum surface potentials (V_S_^min^ and V_S_^max^), average positive and negative surface potentials (V_S_^+^ and V_S_^−^), their variances (σ_+_^2^ and σ_−_^2^), the total variance (σ_T_^2^), the average deviation from the mean surface potential (π), balance (η_S_) and indicator of electrostatic interactive tendencies η_S_σ_T_^2^ obtained for the compounds studied.

Molecular System	X	V_S_^max^ [kJ/mol]	V_S_^min^ [kJ/mol]	V_S_^+^ [kJ/mol]	σ_+_^2^ [kJ/mol]	V_S_^−^ [kJ/mol]	σ_−_^2^ [kJ/mol]	π [kJ/mol]	η_S_ [-]	σ_T_^2^ [kJ/mol]	η_S_σ_T_^2^ [kJ/mol]
AB	H	50.14	−97.09	18.89	0.06	−31.25	0.40	19.45	0.12	0.47	0.06
tClAB	Cl	67.09	−87.23	23.89	0.11	−23.06	0.21	21.95	0.23	0.32	0.07
tBrAB	Br	85.01	−103.20	28.06	0.15	−28.06	0.31	26.81	0.22	0.46	0.10
tIAB	I	63.76	−82.23	22.09	0.10	−20.84	0.16	20.56	0.24	0.46	0.11

**Table 8:** Topological parameters of ρ(r) for the interactions in tIAB, tBrAB, tClAB and AB (electron density at bond critical point, BCP, (ρ_BCP_(r)), its Laplacian Δρ_BCP_(r), the potential electron energy density (*V*_BCP_(r)), the kinetic electron energy density (*G*_BCP_(r)), the total electron energy density (H_BCP_(r)), the bonding energy according to Espinosa−Molins−Lecomte (E_HBE_), Matta (E_HBM_)_,_ Emamian (E_HBEM_), Afonin (E_HA_) and Kuznetsov (E_XXK_), calculated at the B3LYP/TZP with D3.

Molecular System	Type of Interaction	Interaction	ρ_BCP_(r) [a.u.]	Δρ_BCP_(r) [a.u.]	V_BCP_ [a.u.]	G_BCP_ [a.u.]	H_BCP_ [a.u.]	E_HBE_ [kJ/mol]	E_HBM_ [kJ/mol]	E_HBEM_ [kJ/mol]	E_HBA_ [kJ/mol]	E_XXK_ [kJ/mol]
tAB	intra	H9 ⋯ H25	0.0128	0.0459	−0.0074	0.0095	0.0020	−9.79	−10.67	−8.84	−5.88	-
	inter	N3 ⋯ H67, N24 ⋯ H43	0.0068	0.0209	−0.0035	0.0044	0.0009	−4.59	−4.96	−3.24	−3.00	-
tClAB	intra	Cl4 ⋯ H56, Cl29 ⋯ H76	0.0028	0.0094	−0.0013	0.0018	0.0005	−1.71	−2.03	0.49	−1.40	-
	intra	Cl4 ⋯ H55, Cl29 ⋯ H75	0.0070	0.0246	−0.0038	0.0050	0.0012	−4.99	−5.63	−3.43	−3.21	-
	intra	Cl4 ⋯ H52, Cl29 ⋯ H72	0.0109	0.0394	−0.0063	0.0081	0.0018	−8.27	−9.12	−7.07	−5.03	-
	inter	Cl29 ⋯ H64, Cl29 ⋯ H54	0.0032	0.0121	−0.0016	0.0023	0.0007	−2.10	−2.59	0.12	−1.61	-
	inter	H69 ⋯ H87	0.0032	0.0097	−0.0016	0.0020	0.0004	−2.10	−2.25	0.12	−1.61	-
	inter	H64 ⋯ H76	0.0035	0.0107	−0.0018	0.0022	0.0004	−2.36	−2.48	−0.16	−1.76	-
	inter	H66 ⋯ H78	0.0045	0.0130	−0.0024	0.0028	0.0004	−3.15	−3.15	−1.09	−2.20	-
	inter	Cl1 ⋯ Cl27	0.0011	0.0033	−0.0004	0.0006	0.0002	−0.53	−0.68	-	-	−3.51
tBrAB	intra	Br4 ⋯ H56, Br32 ⋯ H84	0.0040	0.0125	−0.0018	0.0025	0.0007	−2.36	−2.82	−0.63	−1.76	-
	intra	Br4 ⋯ H55, Br32 ⋯ H83	0.0077	0.0251	−0.0040	0.0051	0.0011	−5.25	−5.74	−4.08	−3.36	-
	intra	Br4 ⋯ H52, Br32 ⋯ H81	0.0111	0.0368	−0.0061	0.0077	0.0015	−8.01	−8.67	−7.25	−4.89	-
	inter	Br4 ⋯ H74, Br4 ⋯ H71	0.0032	0.0115	−0.0015	0.0022	0.0007	−1.97	−2.48	0.12	−1.54	-
	inter	H67 ⋯ H79	0.0022	0.0073	−0.0011	0.0015	0.0004	−1.44	−1.69	1.05	−1.25	-
	inter	H56 ⋯ H74	0.0034	0.0102	−0.0017	0.0021	0.0004	−2.23	−2.37	−0.07	−1.69	-
	inter	H58 ⋯ H75	0.0050	0.0140	−0.0026	0.0031	0.0004	−3.41	−3.49	−1.56	−2.34	-
	inter	Br2 ⋯ Br29	0.0014	0.0037	−0.0005	0.0007	0.0002	−0.66	−0.79	-	-	−6.27
tIAB	intra	I29 ⋯ H77, I4 ⋯ H57	0.0033	0.0099	−0.0013	0.0019	0.0006	−1.71	−2.14	0.03	−1.40	-
	intra	I29 ⋯ H72, I4 ⋯ H52	0.0147	0.0446	−0.0085	0.0098	0.0013	−11.16	−11.04	−10.61	−6.63	-
	intra	H30 ⋯ H17, H58 ⋯ H75	0.0125	0.0467	−0.0076	0.0096	0.0021	−9.8	−10.78	−8.57	−8.87	
	intra	I1 ⋯ I2, I26 ⋯ I27	0.0139	0.0387	−0.0083	0.0090	0.0007	−10.90	−10.14	-	-	−9.38
	intra	I2 ⋯ I3, I27 ⋯ I28	0.0164	0.0420	−0.0097	0.0101	0.0004	−12.73	−11.38	-	-	−9.37
	intra	I3 ⋯ I4, I28 ⋯ I29	0.0170	0.0433	−0.0102	0.0105	0.0003	−10.76	−11.83	-	-	−9.36
	inter	I26 ⋯ H62, I1 ⋯ H82	0.0040	0.0119	−0.0016	0.0023	0.0007	−2.10	−2.59	−0.63	−1.61	-
	inter	N32 ⋯ H51, N7 ⋯ H71	0.0128	0.0428	−0.0082	0.0095	0.0013	−10.76	−10.70	−8.84	−6.41	-

**Table 9:** A comparison of reactivity descriptors (the Highest Occupied Molecular Orbital, HOMO, Lowest Unoccupied Molecular Orbital, LUMO, HOMO-LUMO gap, ΔE_HOMO-LUMO GAP_, chemical potential, μ, absolute electronegativity, χ, absolute hardness η, softness S, electrophilicity index, ω, the electron-accepting power, ω^+^, the electron-donating power, ω^−^, the net electrophilicity, Δω, and the maximum amount of electronic charge, ΔN_max_) calculated at MP2 level.

Molecular System	HOMO [eV]	LUMO [eV]	ΔE_HOMO-LUMO GAP_ [eV]	μ [eV]	χ [eV]	η [eV]	S [1/eV]	ω [eV]	ω_+_ [eV]	ω_−_ [eV]	Δω [eV]	ΔN_max_ [-]
AB	−8.069	3.646	11.715	−2.212	2.212	5.858	0.171	0.835	2.779	0.567	3346	0.378
tClAB	−8.699	2.466	11.165	−3.116	3.116	5.582	0.179	0.870	3.389	0.272	3661	0.558
tBrAB	−7.751	2.234	9.985	−2.758	2.758	4.993	0.200	0.762	3.008	0.250	3258	0.552
tIAB	−8.497	1.420	9.917	−3.538	3.538	4.959	0.202	1.262	3.640	0.102	3742	0.714

**Table 10:** Docking and scoring results obtained for the compounds studied.

Protein	Ligand	Score [kJ/mol]	Total Energy [kJ/mol]	Steric Protein-Ligand [kJ/mol]	Van der Waals [kJ/mol]
3KXN	AB	−49.55	−51.60	−71.55	−25.56
	tClAB	−60.33	−70.14	−93.91	−25.66
	tBrAB	−59.73	−72.18	−98.33	−26.16
	tIAB	−62.24	−77.21	−95.00	−26.24
	Actual ligand—TIBI	−35.98	−51.18	−55.34	−12.92
2MUV	AB	−44.99	−45.55	−70.06	−28.51
	tClAB	−54.16	−60.78	−84.42	−29.73
	tBrAB	−52.48	−59.71	−83.28	−28.70
	tIAB	−51.59	−58.54	−78.64	−30.08
	Actual ligand	−38.95	−43.83	−55.02	−24.44
2MUW	AB	−38.53	−34.96	−59.18	−27.07
	tClAB	−46.77	−53.77	−71.91	−27.16
	tBrAB	−45.59	−50.71	−70.07	−27.10
	tIAB	−46.76	−51.94	−68.63	−26.87
	Actual ligand	−27.94	−21.63	−39.30	−21.16
2LY01	AB	−48.85	−48.40	−72.40	−29.20
	tClAB	−53.23	−59.74	−79.70	−29.88
	tBrAB	−51.13	−58.19	−76.96	−28.75
	tIAB	−52.93	−58.81	−76.71	−29.29
	Actual ligand	−54.39	−54.53	−70.94	−29.55
6M17 small cave	AB	−31.33	−102.85	−143.43	65.76
	tClAB	−3.312	−62.39	−99.57	55.85
	tBrAB	−16.41	−60.90	−94.51	28.07
	tIAB	−33.41	−62.85	−88.02	4.98
	Actual ligand—Amantadine	−57.52	−57.64	−79.48	−12.92
	Actual ligand—Rimantadine	−75.22	−73.04	−100.15	−30.28
6M17 large cave	AB	−69.71	−79.54	−104.80	−28.93
	tClAB	−78.58	−95.92	−120.56	−27.69
	tBrAB	−78.11	−97.58	−117.05	−27.72
	tIAB	−75.25	−95.84	−110.26	−24.87
7QUR	AB	−64.99	−70.66	−99.70	−30.70
	tClAB	−75.84	−87.30	−119.69	−27.31
	tBrAB	−76.26	−89.58	−117.38	−28.66
	tIAB	−75.52	−89.45	−114.43	−28.28
	Actual ligand	−45.73	−53.78	−61.68	−15.63

## Data Availability

Crystallographic information files (CIF) of AB, tClAB, tBrAB and TIAB have been deposited with Cambridge Structural Database Centre (CCDC 1478829, CCDC 1478831, CCDC 1478832, CCDC 1478834).

## Supplementary Materials

The following supporting information can be downloaded at: https://www.mdpi.com/article/10.3390/molecules28010147/s1, Table S1. Atomic coordinates (×104) and equivalent isotropic displacement parameters (Å2 × 103) for AB. U(eq) is defined as one third of the trace of the orthogonalized Uij tensor. Table S2. Bond lengths [Å] and angles [°] for AB. Table S3. Anisotropic displacement parameters (Å2 × 103) for AB. The anisotropic displacement factor exponent takes the form: −2π2[h2 a*2U11 + … + 2 h k a* b* U12]. Table S4. Hydrogen coordinates (×104) and isotropic displacement parameters (Å2 × 103) for AB. Table S5. Torsion angles [°] for AB. Table S6. Atomic coordinates (×104) and equivalent isotropic displacement parameters (Å2 × 103) for tClAB. U(eq) is defined as one third of the trace of the orthogonalized Uij tensor. Table S7. Bond lengths [Å] and angles [°] for tClAB. Table S8. Anisotropic displacement parameters (Å2 × 103) for tClAB. The anisotropic displacement factor exponent takes the form: −2π2[h2 a*2U11 + … + 2 h k a* b* U12]. Table S9. Hydrogen coordinates (×104) and isotropic displacement parameters (Å2 × 103) for tClAB. Table S10. Torsion angles [°] for tClAB. Table S11. Atomic coordinates (×104) and equivalent isotropic displacement parameters (Å2 × 103) for tBrAB. U(eq) is defined as one third of the trace of the orthogonalized Uij tensor. Table S12. Bond lengths [Å] and angles [°] for tBrAB. Table S13. Anisotropic displacement parameters (Å2 × 103) for tBrAB. The anisotropic displacement factor exponent takes the form: −2π2[ h2 a*2U11 + … + 2 h k a* b* U12]. Table S14. Hydrogen coordinates (×104) and isotropic displacement parameters (Å2 × 103) for tBrAB. Table S15. Torsion angles [°] for tBrAB. Table S16. Atomic coordinates (×104) and equivalent isotropic displacement parameters (Å2 × 103) for tIAB. U(eq) is defined as one third of the trace of the orthogonalized Uij tensor. Table S17. Bond lengths [Å] and angles [°] for tIAB. Table S18. Anisotropic displacement parameters (Å2 × 103) for tIAB. The anisotropic displacement factor exponent takes the form: −2π2[ h2 a*2U11 + … + 2 h k a* b* U12]. Table S19. Hydrogen coordinates (×104) and isotropic displacement parameters (Å2 × 103) for tIAB. Table S20. Torsion angles [°] for tIAB.

## Sample Availability

Samples of the compounds are not available from the authors.
