# Supplementary material for: Synthesis and Crystal Structure of Adamantylated 4,5,6,7-Tetrahalogeno-1H-benzimidazoles Novel Multi-Target Ligands (Potential CK2, M2 and SARS-CoV-2 Inhibitors); X-ray/DFT/QTAIM/Hirshfeld Surfaces/Molecular Docking Study
Source: Molecules. 2022 Dec 24;28(1):147. doi: 10.3390/molecules28010147 (PMC9822452; doi:10.3390/molecules28010147)
Supplement: Supplementary file 1 [file molecules-28-00147-s001.zip › molecules-2071054-supplementary.pdf]

Synthesis and Crystal Structure of Adamantylated 4,5,6,7-tetrahalogeno-1*H*-benzimidazoles Novel Multi-Target Ligands (Potential CK2, M2 and SARS-CoV-2 inhibitors). X-ray/DFT/QTAIM/Hirshfeld Surfaces/Molecular Docking Study

Jolanta Natalia Latosińska, Magdalena Latosińska, Andrzej Orzeszko, Jan Krzysztof Maurin

Supplementary

Table S1. Atomic coordinates (x10^4) and equivalent isotropic displacement parameters (Å^{2}x10^3) for AB. U(eq) is defined as one third of the trace of the orthogonalized U^{ij} tensor.

	x	y	z	U(eq)
N(1)	3967(2)	8424(1)	224(1)	42(1)
C(2)	2053(2)	8781(2)	221(1)	49(1)
N(3)	1227(2)	8644(1)	1259(1)	52(1)
C(4)	2710(2)	8156(1)	2020(1)	46(1)
C(5)	2702(3)	7860(2)	3233(1)	63(1)
C(6)	4409(3)	7446(2)	3765(2)	81(1)
C(7)	6126(3)	7349(2)	3117(2)	79(1)
C(8)	6183(2)	7631(2)	1918(1)	57(1)
C(9)	4439(2)	8023(1)	1387(1)	42(1)
C(10)	5272(2)	8464(1)	-790(1)	47(1)
C(11)	5834(2)	6979(1)	-854(1)	43(1)
C(12)	7413(2)	6947(1)	-1803(1)	38(1)
C(13)	9273(2)	7541(1)	-1597(1)	45(1)
C(14)	10863(2)	7450(2)	-2515(1)	53(1)
C(15)	10220(2)	8331(2)	-3740(1)	59(1)
C(16)	8409(2)	7735(2)	-3978(1)	55(1)
C(17)	6810(2)	7825(2)	-3056(1)	47(1)
C(18)	7865(2)	5387(1)	-1746(1)	48(1)
C(19)	9465(2)	5292(2)	-2663(1)	54(1)
C(20)	11284(2)	5887(2)	-2430(2)	59(1)
C(21)	8824(2)	6169(2)	-3894(1)	61(1)

Table S2. Bond lengths [Å] and angles [°] for AB.

N(1)-C(2)	1.3575(17)
N(1)-C(9)	1.3819(16)
N(1)-C(10)	1.4583(16)
C(2)-N(3)	1.3049(19)
C(2)-H(2)	0.951(16)
N(3)-C(4)	1.3871(18)
C(4)-C(5)	1.391(2)
C(4)-C(9)	1.4006(18)
C(5)-C(6)	1.368(3)
C(5)-H(5)	0.9300
C(6)-C(7)	1.396(3)
C(6)-H(6)	0.9300
C(7)-C(8)	1.375(2)
C(7)-H(7)	0.9300
C(8)-C(9)	1.3895(19)
C(8)-H(8)	0.9300
C(10)-C(11)	1.5243(17)
C(10)-H(10A)	0.9700
C(10)-H(10B)	0.9700
C(11)-C(12)	1.5331(17)
C(11)-H(11A)	0.9700
C(11)-H(11B)	0.9700
C(12)-C(18)	1.5374(16)
C(12)-C(17)	1.5388(17)
C(12)-C(13)	1.5404(16)
C(13)-C(14)	1.5264(19)
C(13)-H(13A)	0.9700
C(13)-H(13B)	0.9700
C(14)-C(15)	1.521(2)
C(14)-C(20)	1.528(2)
C(14)-H(14)	0.9800
C(15)-C(16)	1.526(2)
C(15)-H(15A)	0.9700
C(15)-H(15B)	0.9700

C(16)-C(21)	1.531(2)
C(16)-C(17)	1.5342(19)
C(16)-H(16)	0.9800
C(17)-H(17A)	0.9700
C(17)-H(17B)	0.9700
C(18)-C(19)	1.5311(19)
C(18)-H(18A)	0.9700
C(18)-H(18B)	0.9700
C(19)-C(21)	1.527(2)
C(19)-C(20)	1.527(2)
C(19)-H(19)	0.9800
C(20)-H(20A)	0.9700
C(20)-H(20B)	0.9700
C(21)-H(21A)	0.9700
C(21)-H(21B)	0.9700
C(2)-N(1)-C(9)	105.96(11)
C(2)-N(1)-C(10)	127.35(12)
C(9)-N(1)-C(10)	126.68(11)
N(3)-C(2)-N(1)	114.65(13)
N(3)-C(2)-H(2)	125.6(10)
N(1)-C(2)-H(2)	119.7(10)
C(2)-N(3)-C(4)	104.02(11)
N(3)-C(4)-C(5)	130.14(13)
N(3)-C(4)-C(9)	110.20(11)
C(5)-C(4)-C(9)	119.62(13)
C(6)-C(5)-C(4)	118.10(15)
C(6)-C(5)-H(5)	121.0
C(4)-C(5)-H(5)	121.0
C(5)-C(6)-C(7)	121.56(15)
C(5)-C(6)-H(6)	119.2
C(7)-C(6)-H(6)	119.2
C(8)-C(7)-C(6)	121.80(16)
C(8)-C(7)-H(7)	119.1
C(6)-C(7)-H(7)	119.1
C(7)-C(8)-C(9)	116.33(15)
C(7)-C(8)-H(8)	121.8

C(9)-C(8)-H(8)	121.8
N(1)-C(9)-C(8)	132.19(13)
N(1)-C(9)-C(4)	105.16(11)
C(8)-C(9)-C(4)	122.56(13)
N(1)-C(10)-C(11)	112.91(10)
N(1)-C(10)-H(10A)	109.0
C(11)-C(10)-H(10A)	109.0
N(1)-C(10)-H(10B)	109.0
C(11)-C(10)-H(10B)	109.0
H(10A)-C(10)-H(10B)	107.8
C(10)-C(11)-C(12)	114.77(10)
C(10)-C(11)-H(11A)	108.6
C(12)-C(11)-H(11A)	108.6
C(10)-C(11)-H(11B)	108.6
C(12)-C(11)-H(11B)	108.6
H(11A)-C(11)-H(11B)	107.6
C(11)-C(12)-C(18)	109.05(9)
C(11)-C(12)-C(17)	112.30(10)
C(18)-C(12)-C(17)	108.08(10)
C(11)-C(12)-C(13)	111.11(9)
C(18)-C(12)-C(13)	108.04(10)
C(17)-C(12)-C(13)	108.12(10)
C(14)-C(13)-C(12)	110.80(10)
C(14)-C(13)-H(13A)	109.5
C(12)-C(13)-H(13A)	109.5
C(14)-C(13)-H(13B)	109.5
C(12)-C(13)-H(13B)	109.5
H(13A)-C(13)-H(13B)	108.1
C(15)-C(14)-C(13)	109.13(11)
C(15)-C(14)-C(20)	109.92(12)
C(13)-C(14)-C(20)	109.73(12)
C(15)-C(14)-H(14)	109.3
C(13)-C(14)-H(14)	109.3
C(20)-C(14)-H(14)	109.3
C(14)-C(15)-C(16)	109.73(11)
C(14)-C(15)-H(15A)	109.7

C(16)-C(15)-H(15A)	109.7
C(14)-C(15)-H(15B)	109.7
C(16)-C(15)-H(15B)	109.7
H(15A)-C(15)-H(15B)	108.2
C(15)-C(16)-C(21)	109.52(13)
C(15)-C(16)-C(17)	109.04(11)
C(21)-C(16)-C(17)	109.58(12)
C(15)-C(16)-H(16)	109.6
C(21)-C(16)-H(16)	109.6
C(17)-C(16)-H(16)	109.6
C(16)-C(17)-C(12)	110.61(11)
C(16)-C(17)-H(17A)	109.5
C(12)-C(17)-H(17A)	109.5
C(16)-C(17)-H(17B)	109.5
C(12)-C(17)-H(17B)	109.5
H(17A)-C(17)-H(17B)	108.1
C(19)-C(18)-C(12)	111.17(10)
C(19)-C(18)-H(18A)	109.4
C(12)-C(18)-H(18A)	109.4
C(19)-C(18)-H(18B)	109.4
C(12)-C(18)-H(18B)	109.4
H(18A)-C(18)-H(18B)	108.0
C(21)-C(19)-C(20)	109.69(12)
C(21)-C(19)-C(18)	109.12(12)
C(20)-C(19)-C(18)	109.46(11)
C(21)-C(19)-H(19)	109.5
C(20)-C(19)-H(19)	109.5
C(18)-C(19)-H(19)	109.5
C(19)-C(20)-C(14)	109.16(11)
C(19)-C(20)-H(20A)	109.8
C(14)-C(20)-H(20A)	109.8
C(19)-C(20)-H(20B)	109.8
C(14)-C(20)-H(20B)	109.8
H(20A)-C(20)-H(20B)	108.3
C(19)-C(21)-C(16)	109.47(11)
C(19)-C(21)-H(21A)	109.8

C(16)-C(21)-H(21A)	109.8
C(19)-C(21)-H(21B)	109.8
C(16)-C(21)-H(21B)	109.8
H(21A)-C(21)-H(21B)	108.2

Symmetry transformations used to generate equivalent atoms:

Table S3. Anisotropic displacement parameters ($Å^2x10^3$) for AB. The anisotropic displacement factor exponent takes the form: $-2\pi^2[h^2 a^{*2}U^{11} + ... + 2 h k a^* b^* U^{12}]$

	U^{11}	U^{22}	U^{33}	U^{23}	U^{13}	U^{12}
N(1)	42(1)	41(1)	47(1)	-19(1)	1(1)	-1(1)
C(2)	43(1)	51(1)	58(1)	-24(1)	-7(1)	-1(1)
N(3)	40(1)	57(1)	64(1)	-26(1)	2(1)	-4(1)
C(4)	47(1)	41(1)	52(1)	-20(1)	5(1)	-5(1)
C(5)	71(1)	67(1)	53(1)	-24(1)	12(1)	-2(1)
C(6)	100(1)	94(1)	50(1)	-29(1)	-11(1)	16(1)
C(7)	76(1)	98(1)	69(1)	-38(1)	-26(1)	25(1)
C(8)	49(1)	63(1)	64(1)	-29(1)	-8(1)	11(1)
C(9)	44(1)	36(1)	48(1)	-18(1)	0(1)	-1(1)
C(10)	55(1)	40(1)	47(1)	-17(1)	8(1)	-4(1)
C(11)	48(1)	37(1)	44(1)	-15(1)	3(1)	-5(1)
C(12)	42(1)	34(1)	38(1)	-13(1)	-2(1)	-2(1)
C(13)	45(1)	44(1)	49(1)	-19(1)	-7(1)	-1(1)
C(14)	40(1)	54(1)	67(1)	-25(1)	0(1)	-6(1)
C(15)	64(1)	49(1)	58(1)	-15(1)	17(1)	-9(1)
C(16)	66(1)	59(1)	37(1)	-13(1)	-1(1)	0(1)
C(17)	48(1)	50(1)	43(1)	-15(1)	-8(1)	2(1)
C(18)	55(1)	35(1)	53(1)	-16(1)	4(1)	-4(1)
C(19)	59(1)	41(1)	65(1)	-26(1)	7(1)	0(1)
C(20)	48(1)	57(1)	71(1)	-24(1)	1(1)	8(1)
C(21)	67(1)	71(1)	56(1)	-36(1)	9(1)	-8(1)

Table S4. Hydrogen coordinates (x10^4) and isotropic displacement parameters (Å^{2}x10^3) for AB.

	x	y	z	U(eq)
H(2)	1440(20)	9142(17)	-507(14)	59
H(5)	1569	7940	3669	76
H(6)	4425	7225	4577	97
H(7)	7262	7085	3507	94
H(8)	7327	7563	1487	69
H(10A)	6424	8924	-747	57
H(10B)	4663	9041	-1507	57
H(11A)	4701	6577	-1004	51
H(11B)	6264	6365	-92	51
H(13A)	9027	8533	-1643	54
H(13B)	9686	6996	-813	54
H(14)	12029	7835	-2368	63
H(15A)	11233	8297	-4328	70
H(15B)	9961	9324	-3792	70
H(16)	8003	8300	-4770	67
H(17A)	5648	7460	-3208	57
H(17B)	6538	8819	-3116	57
H(18A)	8256	4815	-963	57
H(18B)	6716	4997	-1888	57
H(19)	9726	4288	-2607	64
H(20A)	12305	5825	-3006	71
H(20B)	11700	5327	-1650	71
H(21A)	7676	5786	-4047	73
H(21B)	9824	6109	-4483	73

Table S5. Torsion angles [°] for AB.

C(9)-N(1)-C(2)-N(3)	-0.56(15)
C(10)-N(1)-C(2)-N(3)	179.94(11)
N(1)-C(2)-N(3)-C(4)	0.10(15)
C(2)-N(3)-C(4)-C(5)	177.83(14)
C(2)-N(3)-C(4)-C(9)	0.40(14)
N(3)-C(4)-C(5)-C(6)	-177.13(16)
C(9)-C(4)-C(5)-C(6)	0.1(2)
C(4)-C(5)-C(6)-C(7)	1.3(3)
C(5)-C(6)-C(7)-C(8)	-1.5(3)
C(6)-C(7)-C(8)-C(9)	0.2(3)
C(2)-N(1)-C(9)-C(8)	-175.94(14)
C(10)-N(1)-C(9)-C(8)	3.6(2)
C(2)-N(1)-C(9)-C(4)	0.75(13)
C(10)-N(1)-C(9)-C(4)	-179.74(11)
C(7)-C(8)-C(9)-N(1)	177.41(14)
C(7)-C(8)-C(9)-C(4)	1.2(2)
N(3)-C(4)-C(9)-N(1)	-0.73(14)
C(5)-C(4)-C(9)-N(1)	-178.47(12)
N(3)-C(4)-C(9)-C(8)	176.36(12)
C(5)-C(4)-C(9)-C(8)	-1.4(2)
C(2)-N(1)-C(10)-C(11)	-105.17(15)
C(9)-N(1)-C(10)-C(11)	75.42(15)
N(1)-C(10)-C(11)-C(12)	-171.99(10)
C(10)-C(11)-C(12)-C(18)	178.62(11)
C(10)-C(11)-C(12)-C(17)	-61.61(14)
C(10)-C(11)-C(12)-C(13)	59.64(14)
C(11)-C(12)-C(13)-C(14)	177.69(10)
C(18)-C(12)-C(13)-C(14)	58.10(13)
C(17)-C(12)-C(13)-C(14)	-58.65(13)
C(12)-C(13)-C(14)-C(15)	60.26(14)
C(12)-C(13)-C(14)-C(20)	-60.24(14)
C(13)-C(14)-C(15)-C(16)	-60.65(15)
C(20)-C(14)-C(15)-C(16)	59.73(15)
C(14)-C(15)-C(16)-C(21)	-59.39(15)

C(14)-C(15)-C(16)-C(17)	60.51(15)
C(15)-C(16)-C(17)-C(12)	-60.00(14)
C(21)-C(16)-C(17)-C(12)	59.86(15)
C(11)-C(12)-C(17)-C(16)	-178.60(10)
C(18)-C(12)-C(17)-C(16)	-58.27(14)
C(13)-C(12)-C(17)-C(16)	58.45(13)
C(11)-C(12)-C(18)-C(19)	-178.91(11)
C(17)-C(12)-C(18)-C(19)	58.74(14)
C(13)-C(12)-C(18)-C(19)	-58.04(14)
C(12)-C(18)-C(19)-C(21)	-60.21(14)
C(12)-C(18)-C(19)-C(20)	59.83(15)
C(21)-C(19)-C(20)-C(14)	59.92(15)
C(18)-C(19)-C(20)-C(14)	-59.77(16)
C(15)-C(14)-C(20)-C(19)	-59.78(16)
C(13)-C(14)-C(20)-C(19)	60.24(15)
C(20)-C(19)-C(21)-C(16)	-60.01(15)
C(18)-C(19)-C(21)-C(16)	59.89(15)
C(15)-C(16)-C(21)-C(19)	59.51(15)
C(17)-C(16)-C(21)-C(19)	-60.05(16)

Symmetry transformations used to generate equivalent atoms:

Table S6. Atomic coordinates (x10^4) and equivalent isotropic displacement parameters (Å^{2}x 10^3) for tClAB. U(eq) is defined as one third of the trace of the orthogonalized U^{ij} tensor.

	x	y	z	U(eq)
Cl(1)	1924(1)	9752(1)	-2668(1)	100(1)
Cl(2)	2133(1)	8011(1)	-445(1)	98(1)
Cl(3)	4051(1)	7257(1)	763(1)	96(1)
Cl(4)	5780(1)	8281(1)	-87(1)	72(1)
N(1)	5195(2)	10052(2)	-2421(3)	61(1)
C(2)	4685(3)	10652(3)	-3257(4)	69(1)
N(3)	3797(2)	10537(3)	-3307(3)	74(1)
C(4)	3742(2)	9769(3)	-2359(3)	54(1)
C(5)	2974(2)	9324(3)	-1946(3)	60(1)
C(6)	3077(2)	8560(3)	-988(4)	63(1)
C(7)	3944(3)	8239(3)	-437(3)	61(1)
C(8)	4717(2)	8682(3)	-824(3)	54(1)
C(9)	4604(2)	9446(2)	-1795(3)	50(1)
C(10)	6198(2)	10051(3)	-2249(4)	64(1)
C(11)	6584(2)	9097(3)	-2924(3)	59(1)
C(12)	7630(2)	9102(3)	-2806(3)	51(1)
C(13)	8074(2)	9043(4)	-1437(3)	68(1)
C(14)	9114(3)	9015(4)	-1378(4)	81(1)
C(15)	9429(3)	10057(4)	-1971(5)	83(1)
C(16)	9001(3)	10114(3)	-3347(4)	76(1)
C(17)	7968(3)	10135(3)	-3408(4)	65(1)
C(18)	7928(3)	8082(3)	-3504(4)	68(1)
C(19)	8963(3)	8052(4)	-3452(4)	76(1)
C(20)	9394(3)	8007(4)	-2071(5)	89(1)
C(21)	9275(3)	9093(4)	-4044(4)	83(1)

Table S7. Bond lengths [Å] and angles [°] for tClAB.

Cl(1)-C(5)	1.723(3)
Cl(2)-C(6)	1.728(3)
Cl(3)-C(7)	1.735(3)
Cl(4)-C(8)	1.735(3)
N(1)-C(2)	1.306(5)
N(1)-C(9)	1.386(4)
N(1)-C(10)	1.480(4)
C(2)-N(3)	1.324(5)
C(2)-H(2)	0.9300
N(3)-C(4)	1.382(4)
C(4)-C(5)	1.390(5)
C(4)-C(9)	1.396(4)
C(5)-C(6)	1.369(5)
C(6)-C(7)	1.396(5)
C(7)-C(8)	1.384(5)
C(8)-C(9)	1.379(5)
C(10)-C(11)	1.512(5)
C(10)-H(10A)	0.9700
C(10)-H(10B)	0.9700
C(11)-C(12)	1.545(4)
C(11)-H(11A)	0.9700
C(11)-H(11B)	0.9700
C(12)-C(17)	1.517(5)
C(12)-C(13)	1.522(5)
C(12)-C(18)	1.533(4)
C(13)-C(14)	1.542(5)
C(13)-H(13A)	0.9700
C(13)-H(13B)	0.9700
C(14)-C(15)	1.509(7)
C(14)-C(20)	1.509(7)
C(14)-H(14)	0.9800
C(15)-C(16)	1.521(6)
C(15)-H(15A)	0.9700
C(15)-H(15B)	0.9700

C(16)-C(21)	1.521(6)
C(16)-C(17)	1.531(5)
C(16)-H(16)	0.9800
C(17)-H(17A)	0.9700
C(17)-H(17B)	0.9700
C(18)-C(19)	1.535(5)
C(18)-H(18A)	0.9700
C(18)-H(18B)	0.9700
C(19)-C(21)	1.506(6)
C(19)-C(20)	1.528(6)
C(19)-H(19)	0.9800
C(20)-H(20A)	0.9700
C(20)-H(20B)	0.9700
C(21)-H(21A)	0.9700
C(21)-H(21B)	0.9700
C(2)-N(1)-C(9)	105.8(3)
C(2)-N(1)-C(10)	124.5(3)
C(9)-N(1)-C(10)	129.7(3)
N(1)-C(2)-N(3)	116.9(3)
N(1)-C(2)-H(2)	121.6
N(3)-C(2)-H(2)	121.6
C(2)-N(3)-C(4)	101.6(3)
N(3)-C(4)-C(5)	128.8(3)
N(3)-C(4)-C(9)	110.9(3)
C(5)-C(4)-C(9)	120.3(3)
C(6)-C(5)-C(4)	119.0(3)
C(6)-C(5)-Cl(1)	122.4(3)
C(4)-C(5)-Cl(1)	118.6(3)
C(5)-C(6)-C(7)	120.1(3)
C(5)-C(6)-Cl(2)	119.8(3)
C(7)-C(6)-Cl(2)	120.1(3)
C(8)-C(7)-C(6)	121.8(3)
C(8)-C(7)-Cl(3)	119.3(3)
C(6)-C(7)-Cl(3)	118.9(3)
C(9)-C(8)-C(7)	117.6(3)
C(9)-C(8)-Cl(4)	122.3(3)

C(7)-C(8)-Cl(4)	120.1(3)
C(8)-C(9)-N(1)	134.1(3)
C(8)-C(9)-C(4)	121.2(3)
N(1)-C(9)-C(4)	104.7(3)
N(1)-C(10)-C(11)	112.7(3)
N(1)-C(10)-H(10A)	109.0
C(11)-C(10)-H(10A)	109.0
N(1)-C(10)-H(10B)	109.0
C(11)-C(10)-H(10B)	109.0
H(10A)-C(10)-H(10B)	107.8
C(10)-C(11)-C(12)	113.9(3)
C(10)-C(11)-H(11A)	108.8
C(12)-C(11)-H(11A)	108.8
C(10)-C(11)-H(11B)	108.8
C(12)-C(11)-H(11B)	108.8
H(11A)-C(11)-H(11B)	107.7
C(17)-C(12)-C(13)	108.7(3)
C(17)-C(12)-C(18)	108.1(3)
C(13)-C(12)-C(18)	108.3(3)
C(17)-C(12)-C(11)	111.1(3)
C(13)-C(12)-C(11)	112.1(3)
C(18)-C(12)-C(11)	108.4(3)
C(12)-C(13)-C(14)	109.8(3)
C(12)-C(13)-H(13A)	109.7
C(14)-C(13)-H(13A)	109.7
C(12)-C(13)-H(13B)	109.7
C(14)-C(13)-H(13B)	109.7
H(13A)-C(13)-H(13B)	108.2
C(15)-C(14)-C(20)	109.6(4)
C(15)-C(14)-C(13)	109.6(4)
C(20)-C(14)-C(13)	110.1(4)
C(15)-C(14)-H(14)	109.2
C(20)-C(14)-H(14)	109.2
C(13)-C(14)-H(14)	109.2
C(14)-C(15)-C(16)	109.3(3)
C(14)-C(15)-H(15A)	109.8

C(16)-C(15)-H(15A)	109.8
C(14)-C(15)-H(15B)	109.8
C(16)-C(15)-H(15B)	109.8
H(15A)-C(15)-H(15B)	108.3
C(21)-C(16)-C(15)	109.5(4)
C(21)-C(16)-C(17)	109.3(3)
C(15)-C(16)-C(17)	108.9(3)
C(21)-C(16)-H(16)	109.7
C(15)-C(16)-H(16)	109.7
C(17)-C(16)-H(16)	109.7
C(12)-C(17)-C(16)	111.1(3)
C(12)-C(17)-H(17A)	109.4
C(16)-C(17)-H(17A)	109.4
C(12)-C(17)-H(17B)	109.4
C(16)-C(17)-H(17B)	109.4
H(17A)-C(17)-H(17B)	108.0
C(12)-C(18)-C(19)	111.1(3)
C(12)-C(18)-H(18A)	109.4
C(19)-C(18)-H(18A)	109.4
C(12)-C(18)-H(18B)	109.4
C(19)-C(18)-H(18B)	109.4
H(18A)-C(18)-H(18B)	108.0
C(21)-C(19)-C(20)	108.9(4)
C(21)-C(19)-C(18)	109.5(3)
C(20)-C(19)-C(18)	108.7(3)
C(21)-C(19)-H(19)	109.9
C(20)-C(19)-H(19)	109.9
C(18)-C(19)-H(19)	109.9
C(14)-C(20)-C(19)	109.8(3)
C(14)-C(20)-H(20A)	109.7
C(19)-C(20)-H(20A)	109.7
C(14)-C(20)-H(20B)	109.7
C(19)-C(20)-H(20B)	109.7
H(20A)-C(20)-H(20B)	108.2
C(19)-C(21)-C(16)	110.1(3)
C(19)-C(21)-H(21A)	109.6

C(16)-C(21)-H(21A)	109.6
C(19)-C(21)-H(21B)	109.6
C(16)-C(21)-H(21B)	109.6
H(21A)-C(21)-H(21B)	108.2

Table S8. Anisotropic displacement parameters ($Å^2x10^3$) for tClAB. The anisotropic displacement factor exponent takes the form: $-2\pi^2[h^2 a^{*2}U^{11} + ... + 2 h k a^* b^* U^{12}]$

	U^{11}	U^{22}	U^{33}	U^{23}	U^{13}	U^{12}
Cl(1)	47(1)	125(1)	122(1)	1(1)	-1(1)	8(1)
Cl(2)	93(1)	79(1)	137(1)	-15(1)	64(1)	-21(1)
Cl(3)	138(1)	72(1)	87(1)	25(1)	48(1)	19(1)
Cl(4)	72(1)	72(1)	67(1)	-1(1)	-5(1)	22(1)
N(1)	49(2)	57(2)	76(2)	-1(1)	12(1)	-1(1)
C(2)	76(2)	60(2)	73(2)	18(2)	23(2)	0(2)
N(3)	70(2)	76(2)	74(2)	19(2)	7(2)	9(2)
C(4)	55(2)	54(2)	52(2)	0(1)	5(1)	4(1)
C(5)	47(2)	65(2)	67(2)	-15(2)	8(1)	2(1)
C(6)	65(2)	53(2)	76(2)	-17(2)	31(2)	-9(2)
C(7)	90(2)	44(2)	53(2)	-2(1)	23(2)	6(2)
C(8)	58(2)	50(2)	53(2)	-10(1)	2(1)	12(1)
C(9)	48(2)	49(2)	54(2)	-11(1)	11(1)	0(1)
C(10)	46(2)	58(2)	89(2)	-12(2)	14(2)	-4(1)
C(11)	51(2)	62(2)	65(2)	-11(2)	10(1)	-6(1)
C(12)	46(2)	54(2)	52(2)	-7(1)	10(1)	-5(1)
C(13)	61(2)	90(3)	53(2)	4(2)	11(2)	-5(2)
C(14)	61(2)	122(4)	55(2)	1(2)	-6(2)	3(2)
C(15)	50(2)	100(3)	100(3)	-21(2)	13(2)	-16(2)
C(16)	62(2)	73(2)	98(3)	16(2)	29(2)	-8(2)
C(17)	60(2)	65(2)	73(2)	8(2)	17(2)	2(2)
C(18)	62(2)	67(2)	76(2)	-19(2)	14(2)	1(2)
C(19)	66(2)	77(2)	86(3)	-15(2)	18(2)	14(2)
C(20)	66(2)	100(3)	99(3)	22(3)	7(2)	20(2)
C(21)	66(2)	117(4)	72(2)	3(2)	27(2)	9(2)

Table S9. Hydrogen coordinates (x10^4) and isotropic displacement parameters (Å^{2}x 10^3) for tClAB.

	x	y	z	U(eq)
H(2)	4934	11138	-3792	82
H(10A)	6423	10006	-1355	77
H(10B)	6409	10748	-2563	77
H(11A)	6335	9125	-3811	71
H(11B)	6391	8402	-2585	71
H(13A)	7871	8379	-1043	81
H(13B)	7898	9686	-980	81
H(14)	9393	8978	-494	97
H(15A)	10084	10051	-1918	100
H(15B)	9256	10706	-1522	100
H(16)	9204	10789	-3738	91
H(17A)	7791	10790	-2973	78
H(17B)	7690	10180	-4282	78
H(18A)	7646	8103	-4378	82
H(18B)	7728	7410	-3123	82
H(19)	9141	7395	-3903	91
H(20A)	9203	7336	-1680	107
H(20B)	10049	7989	-2022	107
H(21A)	9007	9130	-4924	100
H(21B)	9929	9077	-4011	100

Table S10. Torsion angles [°] for tClAB.

C(9)-N(1)-C(2)-N(3)	-0.6(5)
C(10)-N(1)-C(2)-N(3)	178.6(3)
N(1)-C(2)-N(3)-C(4)	0.9(5)
C(2)-N(3)-C(4)-C(5)	179.1(4)
C(2)-N(3)-C(4)-C(9)	-0.9(4)
N(3)-C(4)-C(5)-C(6)	179.5(3)
C(9)-C(4)-C(5)-C(6)	-0.5(5)
N(3)-C(4)-C(5)-Cl(1)	-0.1(5)
C(9)-C(4)-C(5)-Cl(1)	179.8(2)
C(4)-C(5)-C(6)-C(7)	0.2(5)
Cl(1)-C(5)-C(6)-C(7)	179.8(3)
C(4)-C(5)-C(6)-Cl(2)	179.1(2)
Cl(1)-C(5)-C(6)-Cl(2)	-1.2(4)
C(5)-C(6)-C(7)-C(8)	0.6(5)
Cl(2)-C(6)-C(7)-C(8)	-178.3(2)
C(5)-C(6)-C(7)-Cl(3)	-179.3(3)
Cl(2)-C(6)-C(7)-Cl(3)	1.8(4)
C(6)-C(7)-C(8)-C(9)	-1.1(5)
Cl(3)-C(7)-C(8)-C(9)	178.9(2)
C(6)-C(7)-C(8)-Cl(4)	178.6(2)
Cl(3)-C(7)-C(8)-Cl(4)	-1.5(4)
C(7)-C(8)-C(9)-N(1)	179.9(3)
Cl(4)-C(8)-C(9)-N(1)	0.3(5)
C(7)-C(8)-C(9)-C(4)	0.7(4)
Cl(4)-C(8)-C(9)-C(4)	-178.9(2)
C(2)-N(1)-C(9)-C(8)	-179.4(4)
C(10)-N(1)-C(9)-C(8)	1.6(6)
C(2)-N(1)-C(9)-C(4)	0.0(4)
C(10)-N(1)-C(9)-C(4)	-179.1(3)
N(3)-C(4)-C(9)-C(8)	-180.0(3)
C(5)-C(4)-C(9)-C(8)	0.1(5)
N(3)-C(4)-C(9)-N(1)	0.6(4)
C(5)-C(4)-C(9)-N(1)	-179.3(3)
C(2)-N(1)-C(10)-C(11)	-97.4(4)

C(9)-N(1)-C(10)-C(11)	81.5(5)
N(1)-C(10)-C(11)-C(12)	177.6(3)
C(10)-C(11)-C(12)-C(17)	-62.6(4)
C(10)-C(11)-C(12)-C(13)	59.3(4)
C(10)-C(11)-C(12)-C(18)	178.8(3)
C(17)-C(12)-C(13)-C(14)	-58.5(4)
C(18)-C(12)-C(13)-C(14)	58.8(4)
C(11)-C(12)-C(13)-C(14)	178.3(3)
C(12)-C(13)-C(14)-C(15)	60.4(4)
C(12)-C(13)-C(14)-C(20)	-60.2(4)
C(20)-C(14)-C(15)-C(16)	60.1(4)
C(13)-C(14)-C(15)-C(16)	-60.8(4)
C(14)-C(15)-C(16)-C(21)	-59.4(4)
C(14)-C(15)-C(16)-C(17)	60.0(4)
C(13)-C(12)-C(17)-C(16)	58.9(4)
C(18)-C(12)-C(17)-C(16)	-58.5(4)
C(11)-C(12)-C(17)-C(16)	-177.3(3)
C(21)-C(16)-C(17)-C(12)	59.8(4)
C(15)-C(16)-C(17)-C(12)	-59.7(4)
C(17)-C(12)-C(18)-C(19)	58.1(4)
C(13)-C(12)-C(18)-C(19)	-59.6(4)
C(11)-C(12)-C(18)-C(19)	178.6(3)
C(12)-C(18)-C(19)-C(21)	-59.1(4)
C(12)-C(18)-C(19)-C(20)	59.6(5)
C(15)-C(14)-C(20)-C(19)	-60.5(5)
C(13)-C(14)-C(20)-C(19)	60.1(5)
C(21)-C(19)-C(20)-C(14)	59.9(5)
C(18)-C(19)-C(20)-C(14)	-59.2(5)
C(20)-C(19)-C(21)-C(16)	-59.5(4)
C(18)-C(19)-C(21)-C(16)	59.2(5)
C(15)-C(16)-C(21)-C(19)	59.7(4)
C(17)-C(16)-C(21)-C(19)	-59.5(4)

Table S11. Atomic coordinates ($x10^4$) and equivalent isotropic displacement parameters ($Å^2 x 10^3$) for tBrAB. U(eq) is defined as one third of the trace of the orthogonalized U^{ij} tensor.

	x	y	z	U(eq)
Br(1)	-3048(1)	4891(2)	-2788(2)	103(1)
Br(2)	-2958(1)	3049(2)	-514(2)	102(1)
Br(3)	-1006(2)	2209(2)	776(2)	100(1)
Br(4)	840(1)	3232(1)	-53(1)	77(1)
N(1)	243(7)	5043(9)	-2388(12)	68(3)
C(2)	-58(13)	5596(9)	-3162(12)	84(5)
N(3)	-1103(9)	5539(11)	-3282(12)	81(3)
C(4)	-1210(9)	4802(10)	-2356(12)	63(3)
C(5)	-1949(8)	4375(12)	-1944(13)	66(3)
C(6)	-1891(8)	3618(11)	-1058(14)	70(4)
C(7)	-1081(11)	3279(9)	-485(11)	68(3)
C(8)	-264(8)	3713(9)	-863(10)	59(3)
C(9)	-355(7)	4474(8)	-1813(10)	52(2)
C(10)	1242(8)	5049(12)	-2246(15)	74(3)
C(11)	1628(8)	4131(11)	-2920(12)	63(3)
C(12)	2667(7)	4071(10)	-2737(12)	59(3)
C(13)	3064(10)	3978(14)	-1421(13)	78(4)
C(14)	4105(12)	3916(17)	-1336(15)	90(5)
C(15)	4450(11)	5000(17)	-1881(19)	97(5)
C(16)	4081(11)	5083(15)	-3196(18)	90(5)
C(17)	3074(11)	5127(13)	-3303(15)	77(4)
C(18)	2998(9)	3091(12)	-3475(15)	71(3)
C(19)	3988(12)	3090(14)	-3421(16)	89(5)
C(20)	4351(12)	2963(17)	-2040(20)	101(5)
C(21)	4338(12)	4127(17)	-3898(16)	94(5)

Table S12. Bond lengths [Å] and angles [°] for tBrAB.

Br(1)-C(5)	1.907(13)
Br(2)-C(6)	1.912(11)
Br(3)-C(7)	1.889(12)
Br(4)-C(8)	1.887(11)
N(1)-C(2)	1.130(19)
N(1)-C(9)	1.351(16)
N(1)-C(10)	1.499(16)
C(2)-N(3)	1.57(2)
C(2)-H(2)	0.9300
N(3)-C(4)	1.374(18)
C(4)-C(5)	1.356(18)
C(4)-C(9)	1.414(17)
C(5)-C(6)	1.33(2)
C(6)-C(7)	1.37(2)
C(7)-C(8)	1.45(2)
C(8)-C(9)	1.383(16)
C(10)-C(11)	1.50(2)
C(10)-H(10A)	0.9700
C(10)-H(10B)	0.9700
C(11)-C(12)	1.562(15)
C(11)-H(11A)	0.9700
C(11)-H(11B)	0.9700
C(12)-C(13)	1.48(2)
C(12)-C(18)	1.561(17)
C(12)-C(17)	1.591(19)
C(13)-C(14)	1.57(2)
C(13)-H(13A)	0.9700
C(13)-H(13B)	0.9700
C(14)-C(20)	1.47(3)
C(14)-C(15)	1.57(3)
C(14)-H(14)	0.9800
C(15)-C(16)	1.46(3)
C(15)-H(15A)	0.9700
C(15)-H(15B)	0.9700

C(16)-C(21)	1.48(3)
C(16)-C(17)	1.51(2)
C(16)-H(16)	0.9800
C(17)-H(17A)	0.9700
C(17)-H(17B)	0.9700
C(18)-C(19)	1.49(2)
C(18)-H(18A)	0.9700
C(18)-H(18B)	0.9700
C(19)-C(21)	1.50(3)
C(19)-C(20)	1.53(3)
C(19)-H(19)	0.9800
C(20)-H(20A)	0.9700
C(20)-H(20B)	0.9700
C(21)-H(21A)	0.9700
C(21)-H(21B)	0.9700
C(2)-N(1)-C(9)	114.8(13)
C(2)-N(1)-C(10)	112.6(13)
C(9)-N(1)-C(10)	132.4(12)
N(1)-C(2)-N(3)	110.3(10)
N(1)-C(2)-H(2)	124.9
N(3)-C(2)-H(2)	124.9
C(4)-N(3)-C(2)	99.9(10)
C(5)-C(4)-N(3)	131.8(13)
C(5)-C(4)-C(9)	120.0(12)
N(3)-C(4)-C(9)	108.1(11)
C(6)-C(5)-C(4)	121.4(12)
C(6)-C(5)-Br(1)	123.8(9)
C(4)-C(5)-Br(1)	114.8(11)
C(5)-C(6)-C(7)	121.1(11)
C(5)-C(6)-Br(2)	119.4(11)
C(7)-C(6)-Br(2)	119.4(11)
C(6)-C(7)-C(8)	120.5(11)
C(6)-C(7)-Br(3)	120.8(10)
C(8)-C(7)-Br(3)	118.7(11)
C(9)-C(8)-C(7)	116.5(10)

C(9)-C(8)-Br(4)	124.1(10)
C(7)-C(8)-Br(4)	119.4(9)
N(1)-C(9)-C(8)	132.8(11)
N(1)-C(9)-C(4)	106.8(11)
C(8)-C(9)-C(4)	120.4(11)
N(1)-C(10)-C(11)	113.0(11)
N(1)-C(10)-H(10A)	109.0
C(11)-C(10)-H(10A)	109.0
N(1)-C(10)-H(10B)	109.0
C(11)-C(10)-H(10B)	109.0
H(10A)-C(10)-H(10B)	107.8
C(10)-C(11)-C(12)	114.6(10)
C(10)-C(11)-H(11A)	108.6
C(12)-C(11)-H(11A)	108.6
C(10)-C(11)-H(11B)	108.6
C(12)-C(11)-H(11B)	108.6
H(11A)-C(11)-H(11B)	107.6
C(13)-C(12)-C(18)	108.2(12)
C(13)-C(12)-C(11)	114.4(10)
C(18)-C(12)-C(11)	110.5(10)
C(13)-C(12)-C(17)	107.5(11)
C(18)-C(12)-C(17)	106.0(10)
C(11)-C(12)-C(17)	110.0(11)
C(12)-C(13)-C(14)	110.5(11)
C(12)-C(13)-H(13A)	109.5
C(14)-C(13)-H(13A)	109.5
C(12)-C(13)-H(13B)	109.5
C(14)-C(13)-H(13B)	109.5
H(13A)-C(13)-H(13B)	108.1
C(20)-C(14)-C(13)	108.7(15)
C(20)-C(14)-C(15)	111.7(15)
C(13)-C(14)-C(15)	108.1(15)
C(20)-C(14)-H(14)	109.4
C(13)-C(14)-H(14)	109.4
C(15)-C(14)-H(14)	109.4
C(16)-C(15)-C(14)	108.4(15)

C(16)-C(15)-H(15A)	110.0
C(14)-C(15)-H(15A)	110.0
C(16)-C(15)-H(15B)	110.0
C(14)-C(15)-H(15B)	110.0
H(15A)-C(15)-H(15B)	108.4
C(15)-C(16)-C(21)	110.3(16)
C(15)-C(16)-C(17)	110.0(13)
C(21)-C(16)-C(17)	108.2(15)
C(15)-C(16)-H(16)	109.4
C(21)-C(16)-H(16)	109.4
C(17)-C(16)-H(16)	109.4
C(16)-C(17)-C(12)	111.8(12)
C(16)-C(17)-H(17A)	109.3
C(12)-C(17)-H(17A)	109.3
C(16)-C(17)-H(17B)	109.3
C(12)-C(17)-H(17B)	109.3
H(17A)-C(17)-H(17B)	107.9
C(19)-C(18)-C(12)	111.2(11)
C(19)-C(18)-H(18A)	109.4
C(12)-C(18)-H(18A)	109.4
C(19)-C(18)-H(18B)	109.4
C(12)-C(18)-H(18B)	109.4
H(18A)-C(18)-H(18B)	108.0
C(18)-C(19)-C(21)	112.3(14)
C(18)-C(19)-C(20)	106.4(14)
C(21)-C(19)-C(20)	108.7(16)
C(18)-C(19)-H(19)	109.8
C(21)-C(19)-H(19)	109.8
C(20)-C(19)-H(19)	109.8
C(14)-C(20)-C(19)	109.8(14)
C(14)-C(20)-H(20A)	109.7
C(19)-C(20)-H(20A)	109.7
C(14)-C(20)-H(20B)	109.7
C(19)-C(20)-H(20B)	109.7
H(20A)-C(20)-H(20B)	108.2
C(16)-C(21)-C(19)	112.3(12)

C(16)-C(21)-H(21A)	109.1
C(19)-C(21)-H(21A)	109.1
C(16)-C(21)-H(21B)	109.1
C(19)-C(21)-H(21B)	109.1
H(21A)-C(21)-H(21B)	107.9

Table S13. Anisotropic displacement parameters ($Å^2$x 10^3) for tBrAB. The anisotropic displacement factor exponent takes the form: $-2\pi^2$[h^2 $a^{*2}U^{11}$ + ... + 2 h k a* b* U^{12}]

	U^{11}	U^{22}	U^{33}	U^{23}	U^{13}	U^{12}
Br(1)	61(1)	132(2)	115(2)	11(1)	-1(1)	14(1)
Br(2)	94(1)	92(1)	129(2)	-10(1)	55(1)	-15(1)
Br(3)	141(2)	80(1)	89(1)	26(1)	51(1)	24(1)
Br(4)	81(1)	82(1)	66(1)	1(1)	1(1)	24(1)
N(1)	54(5)	57(6)	94(8)	-12(6)	14(5)	-3(4)
C(2)	172(15)	37(5)	53(6)	21(5)	58(8)	-14(7)
N(3)	74(7)	88(8)	79(7)	19(6)	9(6)	10(6)
C(4)	65(6)	54(6)	68(7)	3(5)	-5(5)	-2(5)
C(5)	49(5)	81(8)	69(7)	-10(6)	15(5)	3(5)
C(6)	65(7)	63(7)	89(9)	-30(7)	39(6)	-9(5)
C(7)	114(11)	51(6)	43(5)	1(4)	28(6)	4(6)
C(8)	74(7)	55(6)	46(5)	-3(4)	-7(5)	17(5)
C(9)	52(5)	50(5)	56(5)	-12(4)	8(4)	3(4)
C(10)	49(6)	88(9)	86(9)	5(7)	18(6)	-6(5)
C(11)	55(6)	70(7)	65(7)	-3(5)	10(5)	-13(5)
C(12)	51(5)	62(6)	66(6)	-9(5)	13(5)	-2(4)
C(13)	74(8)	97(10)	63(7)	7(7)	9(6)	-15(7)
C(14)	82(9)	120(14)	68(8)	7(8)	3(7)	2(8)
C(15)	60(7)	125(14)	107(12)	-32(11)	16(8)	-3(8)
C(16)	78(9)	102(12)	95(11)	26(9)	24(8)	-9(8)
C(17)	77(8)	79(9)	78(8)	8(7)	27(7)	2(6)
C(18)	68(7)	69(8)	77(8)	-19(6)	10(6)	1(5)
C(19)	86(9)	97(11)	82(10)	-32(8)	8(7)	22(8)
C(20)	74(9)	111(13)	114(14)	16(11)	3(9)	21(9)
C(21)	86(10)	127(14)	75(9)	-4(9)	37(8)	2(9)

Table S14. Hydrogen coordinates (x10^4) and isotropic displacement parameters (Å^{2}x 10^3) for tBrAB.

	x	y	z	U(eq)
H(2)	267	6023	-3657	100
H(10A)	1462	5006	-1365	88
H(10B)	1445	5730	-2562	88
H(11A)	1429	4198	-3805	76
H(11B)	1394	3453	-2637	76
H(13A)	2838	3332	-1053	93
H(13B)	2893	4602	-954	93
H(14)	4356	3841	-460	108
H(15A)	4261	5616	-1418	116
H(15B)	5095	4998	-1810	116
H(16)	4306	5743	-3558	108
H(17A)	2838	5197	-4175	92
H(17B)	2893	5762	-2866	92
H(18A)	2804	2420	-3125	86
H(18B)	2736	3131	-4340	86
H(19)	4180	2476	-3900	106
H(20A)	4107	2312	-1710	121
H(20B)	4994	2891	-1964	121
H(21A)	4983	4088	-3838	113
H(21B)	4112	4212	-4772	113

Table S15. Torsion angles [°] for tBrAB.

C(9)-N(1)-C(2)-N(3)	1.8(15)
C(10)-N(1)-C(2)-N(3)	179.0(11)
N(1)-C(2)-N(3)-C(4)	-0.4(15)
C(2)-N(3)-C(4)-C(5)	-180.0(14)
C(2)-N(3)-C(4)-C(9)	-1.1(13)
N(3)-C(4)-C(5)-C(6)	176.5(14)
C(9)-C(4)-C(5)-C(6)	-2(2)
N(3)-C(4)-C(5)-Br(1)	0(2)
C(9)-C(4)-C(5)-Br(1)	-179.3(9)
C(4)-C(5)-C(6)-C(7)	3(2)
Br(1)-C(5)-C(6)-C(7)	179.9(9)
C(4)-C(5)-C(6)-Br(2)	179.9(10)
Br(1)-C(5)-C(6)-Br(2)	-3.4(15)
C(5)-C(6)-C(7)-C(8)	-2.2(18)
Br(2)-C(6)-C(7)-C(8)	-178.8(8)
C(5)-C(6)-C(7)-Br(3)	179.7(10)
Br(2)-C(6)-C(7)-Br(3)	3.1(13)
C(6)-C(7)-C(8)-C(9)	0.2(16)
Br(3)-C(7)-C(8)-C(9)	178.4(8)
C(6)-C(7)-C(8)-Br(4)	179.9(9)
Br(3)-C(7)-C(8)-Br(4)	-1.9(12)
C(2)-N(1)-C(9)-C(8)	178.5(13)
C(10)-N(1)-C(9)-C(8)	2(2)
C(2)-N(1)-C(9)-C(4)	-2.5(15)
C(10)-N(1)-C(9)-C(4)	-179.0(14)
C(7)-C(8)-C(9)-N(1)	179.6(11)
Br(4)-C(8)-C(9)-N(1)	-0.1(17)
C(7)-C(8)-C(9)-C(4)	0.8(16)
Br(4)-C(8)-C(9)-C(4)	-178.9(9)
C(5)-C(4)-C(9)-N(1)	-178.9(12)
N(3)-C(4)-C(9)-N(1)	2.1(14)
C(5)-C(4)-C(9)-C(8)	0.2(18)
N(3)-C(4)-C(9)-C(8)	-178.8(11)
C(2)-N(1)-C(10)-C(11)	-96.2(14)

C(9)-N(1)-C(10)-C(11)	80.4(18)
N(1)-C(10)-C(11)-C(12)	-176.9(11)
C(10)-C(11)-C(12)-C(13)	57.7(16)
C(10)-C(11)-C(12)-C(18)	-180.0(12)
C(10)-C(11)-C(12)-C(17)	-63.3(15)
C(18)-C(12)-C(13)-C(14)	56.0(16)
C(11)-C(12)-C(13)-C(14)	179.6(13)
C(17)-C(12)-C(13)-C(14)	-58.0(16)
C(12)-C(13)-C(14)-C(20)	-59.5(19)
C(12)-C(13)-C(14)-C(15)	61.9(18)
C(20)-C(14)-C(15)-C(16)	57.6(19)
C(13)-C(14)-C(15)-C(16)	-62.0(18)
C(14)-C(15)-C(16)-C(21)	-57.6(17)
C(14)-C(15)-C(16)-C(17)	61.7(19)
C(15)-C(16)-C(17)-C(12)	-60(2)
C(21)-C(16)-C(17)-C(12)	60.5(17)
C(13)-C(12)-C(17)-C(16)	57.3(16)
C(18)-C(12)-C(17)-C(16)	-58.2(16)
C(11)-C(12)-C(17)-C(16)	-177.6(13)
C(13)-C(12)-C(18)-C(19)	-60.0(16)
C(11)-C(12)-C(18)-C(19)	174.1(13)
C(17)-C(12)-C(18)-C(19)	55.0(16)
C(12)-C(18)-C(19)-C(21)	-56.7(17)
C(12)-C(18)-C(19)-C(20)	62.1(18)
C(13)-C(14)-C(20)-C(19)	62.4(19)
C(15)-C(14)-C(20)-C(19)	-57(2)
C(18)-C(19)-C(20)-C(14)	-64.5(19)
C(21)-C(19)-C(20)-C(14)	56.6(19)
C(15)-C(16)-C(21)-C(19)	61.5(19)
C(17)-C(16)-C(21)-C(19)	-58.9(19)
C(18)-C(19)-C(21)-C(16)	58.4(19)
C(20)-C(19)-C(21)-C(16)	-59.0(19)

Table S16. Atomic coordinates (x10^4) and equivalent isotropic displacement parameters ($Å^2$x 10^3) for tIAB. U(eq) is defined as one third of the trace of the orthogonalized U^{ij} tensor.

	x	y	z	U(eq)
I(1)	7007(1)	8686(1)	2478(1)	64(1)
I(2)	11006(2)	6595(1)	2813(1)	78(1)
I(3)	13477(1)	5515(1)	991(1)	72(1)
I(4)	12442(1)	6685(1)	-1009(1)	72(1)
N(1)	8557(13)	8728(8)	-682(5)	48(2)
C(2)	7117(16)	9393(10)	-336(6)	53(2)
N(3)	6920(13)	9288(8)	470(5)	54(2)
C(4)	8326(14)	8445(8)	694(6)	45(2)
C(5)	8736(14)	8016(8)	1493(6)	43(2)
C(6)	10244(14)	7207(8)	1582(6)	45(2)
C(7)	11309(15)	6805(8)	868(6)	45(2)
C(8)	10863(14)	7254(8)	54(6)	43(2)
C(9)	9379(13)	8080(8)	-24(5)	41(2)
C(10)	8891(16)	8703(9)	-1627(6)	49(2)
C(11)	7385(14)	7731(8)	-2264(6)	45(2)
C(12)	7523(12)	7699(7)	-3248(5)	35(2)
C(13)	9612(13)	7300(8)	-3482(6)	41(2)
C(14)	9674(13)	7213(8)	-4472(6)	42(2)
C(15)	9371(14)	8511(9)	-4740(6)	46(2)
C(16)	7282(14)	8911(8)	-4526(6)	45(2)
C(17)	7221(14)	8977(7)	-3524(6)	41(2)
C(18)	5770(13)	6756(8)	-3808(6)	41(2)
C(19)	5824(14)	6694(8)	-4810(6)	45(2)
C(20)	7899(15)	6298(8)	-5017(6)	50(2)
C(21)	5546(15)	7965(9)	-5061(6)	49(2)

Table S17. Bond lengths [Å] and angles [°] for tIAB.

I(1)-C(5)	2.096(9)
I(2)-C(6)	2.109(8)
I(3)-C(7)	2.083(9)
I(4)-C(8)	2.099(8)
N(1)-C(2)	1.352(12)
N(1)-C(9)	1.391(11)
N(1)-C(10)	1.498(10)
C(2)-N(3)	1.286(13)
C(2)-H(2)	0.9300
N(3)-C(4)	1.395(12)
C(4)-C(5)	1.380(12)
C(4)-C(9)	1.408(12)
C(5)-C(6)	1.379(13)
C(6)-C(7)	1.416(13)
C(7)-C(8)	1.414(12)
C(8)-C(9)	1.379(12)
C(10)-C(11)	1.518(12)
C(10)-H(10A)	0.9700
C(10)-H(10B)	0.9700
C(11)-C(12)	1.525(11)
C(11)-H(11A)	0.9700
C(11)-H(11B)	0.9700
C(12)-C(13)	1.539(11)
C(12)-C(17)	1.544(10)
C(12)-C(18)	1.545(11)
C(13)-C(14)	1.518(12)
C(13)-H(13A)	0.9700
C(13)-H(13B)	0.9700
C(14)-C(20)	1.529(12)
C(14)-C(15)	1.561(12)
C(14)-H(14)	0.9800
C(15)-C(16)	1.529(13)
C(15)-H(15A)	0.9700
C(15)-H(15B)	0.9700

C(16)-C(21)	1.526(13)
C(16)-C(17)	1.538(12)
C(16)-H(16)	0.9800
C(17)-H(17A)	0.9700
C(17)-H(17B)	0.9700
C(18)-C(19)	1.538(12)
C(18)-H(18A)	0.9700
C(18)-H(18B)	0.9700
C(19)-C(20)	1.516(13)
C(19)-C(21)	1.519(13)
C(19)-H(19)	0.9800
C(20)-H(20A)	0.9700
C(20)-H(20B)	0.9700
C(21)-H(21A)	0.9700
C(21)-H(21B)	0.9700
C(2)-N(1)-C(9)	106.0(7)
C(2)-N(1)-C(10)	121.7(8)
C(9)-N(1)-C(10)	132.0(8)
N(3)-C(2)-N(1)	115.5(8)
N(3)-C(2)-H(2)	122.3
N(1)-C(2)-H(2)	122.3
C(2)-N(3)-C(4)	104.0(8)
C(5)-C(4)-N(3)	127.6(8)
C(5)-C(4)-C(9)	122.3(9)
N(3)-C(4)-C(9)	110.1(8)
C(6)-C(5)-C(4)	118.1(8)
C(6)-C(5)-I(1)	125.8(6)
C(4)-C(5)-I(1)	116.2(7)
C(5)-C(6)-C(7)	121.1(8)
C(5)-C(6)-I(2)	119.1(6)
C(7)-C(6)-I(2)	119.8(7)
C(8)-C(7)-C(6)	120.0(8)
C(8)-C(7)-I(3)	119.4(7)
C(6)-C(7)-I(3)	120.6(6)
C(9)-C(8)-C(7)	118.6(8)
C(9)-C(8)-I(4)	120.8(6)

C(7)-C(8)-I(4)	120.7(7)
C(8)-C(9)-N(1)	135.7(8)
C(8)-C(9)-C(4)	120.0(8)
N(1)-C(9)-C(4)	104.3(7)
N(1)-C(10)-C(11)	111.4(7)
N(1)-C(10)-H(10A)	109.3
C(11)-C(10)-H(10A)	109.3
N(1)-C(10)-H(10B)	109.3
C(11)-C(10)-H(10B)	109.3
H(10A)-C(10)-H(10B)	108.0
C(10)-C(11)-C(12)	114.9(7)
C(10)-C(11)-H(11A)	108.5
C(12)-C(11)-H(11A)	108.5
C(10)-C(11)-H(11B)	108.5
C(12)-C(11)-H(11B)	108.5
H(11A)-C(11)-H(11B)	107.5
C(11)-C(12)-C(13)	112.5(7)
C(11)-C(12)-C(17)	111.3(7)
C(13)-C(12)-C(17)	108.4(6)
C(11)-C(12)-C(18)	108.6(6)
C(13)-C(12)-C(18)	108.0(7)
C(17)-C(12)-C(18)	107.9(6)
C(14)-C(13)-C(12)	111.6(7)
C(14)-C(13)-H(13A)	109.3
C(12)-C(13)-H(13A)	109.3
C(14)-C(13)-H(13B)	109.3
C(12)-C(13)-H(13B)	109.3
H(13A)-C(13)-H(13B)	108.0
C(13)-C(14)-C(20)	110.6(7)
C(13)-C(14)-C(15)	108.8(7)
C(20)-C(14)-C(15)	107.6(7)
C(13)-C(14)-H(14)	109.9
C(20)-C(14)-H(14)	109.9
C(15)-C(14)-H(14)	109.9
C(16)-C(15)-C(14)	109.3(6)
C(16)-C(15)-H(15A)	109.8

C(14)-C(15)-H(15A)	109.8
C(16)-C(15)-H(15B)	109.8
C(14)-C(15)-H(15B)	109.8
H(15A)-C(15)-H(15B)	108.3
C(21)-C(16)-C(15)	108.8(8)
C(21)-C(16)-C(17)	109.6(7)
C(15)-C(16)-C(17)	110.1(7)
C(21)-C(16)-H(16)	109.4
C(15)-C(16)-H(16)	109.4
C(17)-C(16)-H(16)	109.4
C(16)-C(17)-C(12)	110.4(7)
C(16)-C(17)-H(17A)	109.6
C(12)-C(17)-H(17A)	109.6
C(16)-C(17)-H(17B)	109.6
C(12)-C(17)-H(17B)	109.6
H(17A)-C(17)-H(17B)	108.1
C(19)-C(18)-C(12)	110.8(6)
C(19)-C(18)-H(18A)	109.5
C(12)-C(18)-H(18A)	109.5
C(19)-C(18)-H(18B)	109.5
C(12)-C(18)-H(18B)	109.5
H(18A)-C(18)-H(18B)	108.1
C(20)-C(19)-C(21)	109.3(7)
C(20)-C(19)-C(18)	109.6(7)
C(21)-C(19)-C(18)	109.5(7)
C(20)-C(19)-H(19)	109.5
C(21)-C(19)-H(19)	109.5
C(18)-C(19)-H(19)	109.5
C(19)-C(20)-C(14)	110.1(7)
C(19)-C(20)-H(20A)	109.6
C(14)-C(20)-H(20A)	109.6
C(19)-C(20)-H(20B)	109.6
C(14)-C(20)-H(20B)	109.6
H(20A)-C(20)-H(20B)	108.1
C(19)-C(21)-C(16)	109.9(7)
C(19)-C(21)-H(21A)	109.7

C(16)-C(21)-H(21A)	109.7
C(19)-C(21)-H(21B)	109.7
C(16)-C(21)-H(21B)	109.7
H(21A)-C(21)-H(21B)	108.2

Table S18. Anisotropic displacement parameters ($Å^2$x 10^3) for tIAB. The anisotropic displacement factor exponent takes the form: $-2\pi^2[h^2 a^{*2}U^{11} + ... + 2 h k a^* b^* U^{12}]$

	U^{11}	U^{22}	U^{33}	U^{23}	U^{13}	U^{12}
I(1)	65(1)	89(1)	41(1)	2(1)	24(1)	8(1)
I(2)	103(1)	96(1)	44(1)	29(1)	14(1)	26(1)
I(3)	80(1)	76(1)	68(1)	22(1)	16(1)	32(1)
I(4)	78(1)	102(1)	49(1)	19(1)	28(1)	45(1)
N(1)	56(5)	62(4)	28(3)	11(3)	10(3)	12(3)
C(2)	54(6)	64(5)	44(5)	9(4)	5(4)	25(4)
N(3)	51(5)	72(5)	42(4)	8(4)	12(3)	24(4)
C(4)	46(5)	53(5)	33(4)	4(3)	6(3)	5(4)
C(5)	43(5)	52(4)	34(4)	5(3)	10(3)	3(3)
C(6)	49(5)	55(5)	29(4)	11(3)	3(3)	-2(4)
C(7)	47(5)	48(4)	42(5)	10(3)	5(4)	11(4)
C(8)	44(5)	55(5)	31(4)	6(3)	11(3)	9(4)
C(9)	41(4)	48(4)	33(4)	6(3)	8(3)	8(3)
C(10)	57(5)	64(5)	28(4)	13(4)	11(4)	1(4)
C(11)	44(5)	56(5)	34(4)	13(3)	5(3)	-2(4)
C(12)	37(4)	38(4)	32(4)	12(3)	8(3)	4(3)
C(13)	37(4)	42(4)	45(5)	14(3)	6(3)	4(3)
C(14)	36(4)	46(4)	45(5)	5(3)	15(3)	4(3)
C(15)	46(5)	58(5)	38(4)	18(4)	11(4)	-7(4)
C(16)	52(5)	47(4)	40(4)	22(3)	7(4)	7(4)
C(17)	48(5)	37(4)	39(4)	10(3)	10(3)	4(3)
C(18)	34(4)	50(4)	40(4)	14(3)	8(3)	-2(3)
C(19)	42(5)	53(5)	37(4)	3(3)	5(3)	-9(4)
C(20)	55(6)	49(4)	44(5)	-1(4)	14(4)	1(4)
C(21)	42(5)	70(6)	39(4)	19(4)	4(4)	9(4)

Table S19. Hydrogen coordinates (x10^4) and isotropic displacement parameters ($Å^2$x 10^3) for tIAB.

	x	y	z	U(eq)
H(2)	6323	9892	-659	64
H(10A)	10306	8526	-1677	59
H(10B)	8705	9512	-1790	59
H(11A)	5980	7879	-2173	53
H(11B)	7637	6922	-2113	53
H(13A)	10732	7895	-3149	49
H(13B)	9827	6500	-3308	49
H(14)	11012	6950	-4604	50
H(15A)	10490	9113	-4414	56
H(15B)	9403	8470	-5372	56
H(16)	7090	9727	-4691	54
H(17A)	5895	9236	-3393	49
H(17B)	8311	9586	-3184	49
H(18A)	5932	5942	-3649	49
H(18B)	4434	6996	-3678	49
H(19)	4700	6094	-5154	54
H(20A)	7940	6267	-5648	60
H(20B)	8064	5475	-4877	60
H(21A)	4208	8217	-4941	59
H(21B)	5573	7929	-5694	59

Table S20. Torsion angles [°] for tIAB.

C(9)-N(1)-C(2)-N(3)	1.5(12)
C(10)-N(1)-C(2)-N(3)	176.8(9)
N(1)-C(2)-N(3)-C(4)	-1.5(12)
C(2)-N(3)-C(4)-C(5)	179.5(10)
C(2)-N(3)-C(4)-C(9)	0.9(11)
N(3)-C(4)-C(5)-C(6)	-178.0(9)
C(9)-C(4)-C(5)-C(6)	0.4(13)
N(3)-C(4)-C(5)-I(1)	1.2(13)
C(9)-C(4)-C(5)-I(1)	179.6(7)
C(4)-C(5)-C(6)-C(7)	-1.4(13)
I(1)-C(5)-C(6)-C(7)	179.5(7)
C(4)-C(5)-C(6)-I(2)	177.3(6)
I(1)-C(5)-C(6)-I(2)	-1.8(10)
C(5)-C(6)-C(7)-C(8)	1.3(13)
I(2)-C(6)-C(7)-C(8)	-177.3(7)
C(5)-C(6)-C(7)-I(3)	-176.9(7)
I(2)-C(6)-C(7)-I(3)	4.4(10)
C(6)-C(7)-C(8)-C(9)	-0.2(13)
I(3)-C(7)-C(8)-C(9)	178.1(6)
C(6)-C(7)-C(8)-I(4)	179.4(6)
I(3)-C(7)-C(8)-I(4)	-2.3(10)
C(7)-C(8)-C(9)-N(1)	178.5(10)
I(4)-C(8)-C(9)-N(1)	-1.2(15)
C(7)-C(8)-C(9)-C(4)	-0.8(13)
I(4)-C(8)-C(9)-C(4)	179.6(6)
C(2)-N(1)-C(9)-C(8)	179.9(11)
C(10)-N(1)-C(9)-C(8)	5.3(18)
C(2)-N(1)-C(9)-C(4)	-0.8(10)
C(10)-N(1)-C(9)-C(4)	-175.4(9)
C(5)-C(4)-C(9)-C(8)	0.7(14)
N(3)-C(4)-C(9)-C(8)	179.4(8)
C(5)-C(4)-C(9)-N(1)	-178.7(8)
N(3)-C(4)-C(9)-N(1)	-0.1(10)
C(2)-N(1)-C(10)-C(11)	-85.1(11)

C(9)-N(1)-C(10)-C(11)	88.8(12)
N(1)-C(10)-C(11)-C(12)	176.2(8)
C(10)-C(11)-C(12)-C(13)	65.8(10)
C(10)-C(11)-C(12)-C(17)	-56.1(10)
C(10)-C(11)-C(12)-C(18)	-174.7(8)
C(11)-C(12)-C(13)-C(14)	177.2(7)
C(17)-C(12)-C(13)-C(14)	-59.4(8)
C(18)-C(12)-C(13)-C(14)	57.4(8)
C(12)-C(13)-C(14)-C(20)	-57.9(9)
C(12)-C(13)-C(14)-C(15)	60.1(8)
C(13)-C(14)-C(15)-C(16)	-59.3(9)
C(20)-C(14)-C(15)-C(16)	60.6(9)
C(14)-C(15)-C(16)-C(21)	-60.7(9)
C(14)-C(15)-C(16)-C(17)	59.4(9)
C(21)-C(16)-C(17)-C(12)	60.1(9)
C(15)-C(16)-C(17)-C(12)	-59.6(9)
C(11)-C(12)-C(17)-C(16)	-177.8(7)
C(13)-C(12)-C(17)-C(16)	58.0(9)
C(18)-C(12)-C(17)-C(16)	-58.8(9)
C(11)-C(12)-C(18)-C(19)	179.5(7)
C(13)-C(12)-C(18)-C(19)	-58.3(9)
C(17)-C(12)-C(18)-C(19)	58.8(9)
C(12)-C(18)-C(19)-C(20)	60.1(9)
C(12)-C(18)-C(19)-C(21)	-59.8(9)
C(21)-C(19)-C(20)-C(14)	61.2(10)
C(18)-C(19)-C(20)-C(14)	-58.7(9)
C(13)-C(14)-C(20)-C(19)	58.0(10)
C(15)-C(14)-C(20)-C(19)	-60.8(9)
C(20)-C(19)-C(21)-C(16)	-60.4(10)
C(18)-C(19)-C(21)-C(16)	59.6(10)
C(15)-C(16)-C(21)-C(19)	60.5(9)
C(17)-C(16)-C(21)-C(19)	-59.9(10)
